# CLAG: an unsupervised non hierarchical clustering algorithm handling biological data

**DOI:** 10.1186/1471-2105-13-194

**Published:** 2012-08-08

**Authors:** Linda Dib, Alessandra Carbone

**Affiliations:** 1, UPMC, UMR7238, Génomique Analytique, 15 rue de l’Ecole de Médecine, F-75006 Paris, France; 2, CNRS, UMR7238, Laboratoire de Génomique des Microorganismes, F-75006 Paris, France

## Abstract

**Background:**

Searching for similarities in a set of biological data is intrinsically difficult due to possible data points that should not be clustered, or that should group within several clusters. Under these hypotheses, hierarchical agglomerative clustering is not appropriate. Moreover, if the dataset is not known enough, like often is the case, supervised classification is not appropriate either.

**Results:**

CLAG (for CLusters AGgregation) is an unsupervised non hierarchical clustering algorithm designed to cluster a large variety of biological data and to provide a clustered matrix and numerical values indicating cluster strength. CLAG clusterizes correlation matrices for residues in protein families, gene-expression and miRNA data related to various cancer types, sets of species described by multidimensional vectors of characters, binary matrices. It does not ask to all data points to cluster and it converges yielding the same result at each run. Its simplicity and speed allows it to run on reasonably large datasets.

**Conclusions:**

CLAG can be used to investigate the cluster structure present in biological datasets and to identify its underlying graph. It showed to be more informative and accurate than several known clustering methods, as hierarchical agglomerative clustering, *k*-means, fuzzy c-means, model-based clustering, affinity propagation clustering, and not to suffer of the convergence problem proper to this latter.

## Background

Clustering of biological data often requires to look for the proximity of few data points within a large dataset with the purpose to group together only those that satisfy the same set of constraints, possibly resulting from the same functional origins, or that have undergone the same evolutionary pressures. This is the case for amino acids in proteins, where one expects few of the residues to account for the structural stability of the protein or for its functional activity. For these biological problems, the number of expected clusters is unknown and classification approaches, known as unsupervised, are expected to unravel hidden structures in the data.

A common approach to clustering is the simple unsupervised *k*-means clustering technique [1]. It starts with a random selection of *k* samples in the dataset and iteratively creates clusters of data points around the *k* samples by adding new data points to the *k*-centers in such a way that the sum of squared errors between data points and their nearest centers is small. *k*-means clustering is sensitive to the initial selection of data points and it needs to be re-run many times in the attempt to find a satisfiable solution. If *k* is small and there are good chances that at least one random selection of data points will be close to a good solution, *k*-means is an interesting technique to try. Otherwise, the ideal approach would be to simultaneously consider all data points in the set and find, with some well-designed criteria, appropriate candidates for cluster generation [2,3]. We propose a method that tries all data points, that is multidimensional vectors of characters, as generators and extends them by properly identifying data points in the set that share with the generator similar values for the same set of characters and display differences on a complementary set of characters. Through an appropriate discretization of the space of distances, the method always provides a clustering solution and this latter is unique. Depending on the strength of the clusters, measured by the number of similar characters, the method aggregates clusters whenever they share some data points. Aggregates are expected to be biologically significant.

CLAG, for CLusters AGgregation, is an unsupervised non hierarchical clustering algorithm that handles non uniform distributions of values in order to zoom in dense sets of character values, parameterizes data points proximity, and outputs a graph of similarity between data points as well as a clustered matrix.

Important work on clustering a restricted number of datapoints [4-14] or datapoints that might belong to several clusters [15,16] has been previously developed. CLAG is compared to several known clustering methods, as hierarchical agglomerative clustering and *k*-means, and in particular to fuzzy c-means, model-based clustering and affinity propagation clustering. It proves to be informative and accurate, not to suffer of convergence problems proper to some of the methods, and to perform well with multidimensional data.

## Results and discussion

### Clustering algorithm and aggregation

Let us consider a set N of *N* elements and a set E, called *environment*, of *M* characters. To each element we associate a vector of *M* character values, and we consider a *M* × *N* matrix *A* describing the full set of elements in N. Characters can have different nature or might be the *N* elements themselves. In this latter case, the matrix entries might correspond to correlation values or to distances between elements. Without loss of generality, we assume the matrix entries to be reals and we renormalize them in the interval [0,1]. Let *Δ* be a parameter, ranging from 0 to 1, that modulates the proximity between elements. Based on *Δ*, we shall determine if two elements V,Z∈N are close (or similar) with respect to the environment and, whenever possible, if they are symmetric. The idea is to look at the distribution of matrix entries and analyse for each pair of elements in N (that is, for each pairs of columns in the matrix) the localization of all corresponding pairs of matrix entries within the distribution. A measure of closeness between entries is introduced (based on the discretization of the distribution into quantiles grids) and it is used to define the proximity of two elements in N. Then, we define two scores, *environmental* and *symmetric scores*, for pairs of elements in N providing, in this way, a numerical criteria to evaluate clusters’ strength.

#### Entries distributions and grids

The *M*·*N* entries of the matrix are first analyzed by looking at their distribution and dividing it into *Δ*-quantiles (by default, *Δ* is divisible by 0.05), that is subsets of the distribution containing 100·*Δ*% of entries. We denote with *Δ*-*quantile*(S), the *Δ*-quantile computed starting at entry *S* of the distribution. To each *Δ*-quantile, we associate an *interval* which is defined by the minimum *S*_1_ and the maximum *S*_2_ entries within the *Δ*-quantile. The length of the interval is |*S*_2_ − *S*_1_|.

**Figure 1:**
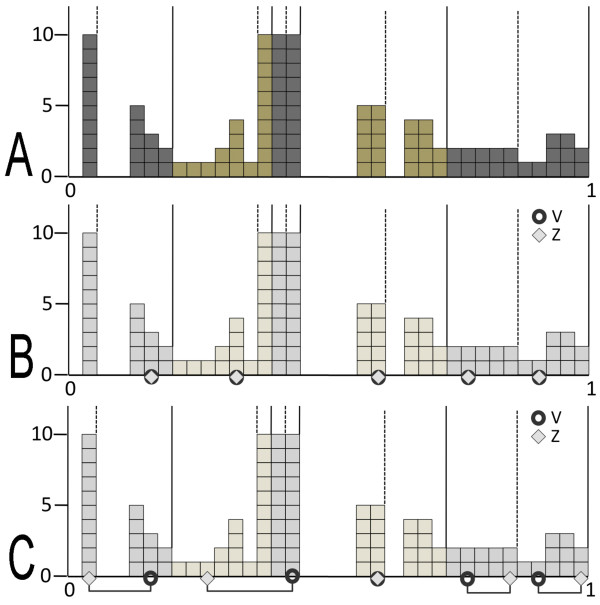
**Entries distribution and grids.** Toy example based on a matrix of 100 entries with environment E of size |*M*| = 5. **A:** the distribution of entries, partitioned with a 0-grid (solid lines) and with a 1-grid (dashed lines). Alternated grey and green colors are used to identify quantile regions. Grids are defined with *Δ* = .20, and therefore, each quantile region contains exactly 20 entries. **B:** elements V,Z∈N, represented by 5 character values in the matrix, are plot in the distribution: 5 circles and 5 diamonts represent *V * and *Z*, respectively. For each X∈E, *A*(*V*,*X*) = *A*(*Z*,*X*) and this implies an environmental score *S*_*env*_(*V*,*Z*) = 1. **C:** contrary to B, here the 5 pairs of entries, *A*(*V*,*X*) and *A*(*Z*,*X*), are not necessarily equal nor highly similar (that is, belonging to the same quantile) and they are indicated by a straight line linking entries positions. *S*_*env*_(*V*,*Z*) is expected not to be maximal.

We discretize the entries distribution with the help of two shifted grids of intervals that will be used to easily define entries closeness. Namely, a *0-grid* is defined by segmenting the distribution in *Δ*-quantiles from the minimal entry of the distribution, and a *1-grid* is defined by segmenting the distribution from the end of the first $\frac{\Delta }{2}$-quantile. The successive intervals of the 0-grid are denoted $I_{i}^{0}$, and those for the 1-grid are denoted $I_{i}^{1}$. Notice that the first and the last intervals of the 1-grid correspond to $\frac{\Delta }{2}$-quantiles. See Figure 1A for an example of distribution and grids.

We say that a distribution of scores is *heterogeneous* in a grid if there are intervals of the grid whose lengths are greater than *μ* + *σ*, where *μ* is the mean of the lengths of the intervals for the entire grid and *σ* is the standard deviation of the distribution of lengths. To avoid having very large and very small intervals associated to *Δ*-quantiles for the same distribution, possibly due to very sparse or very concentrated entries along the distribution, we refine the 0- and 1-grids by cutting in half each interval in them which is larger than *μ* + *σ* and redefine the 0- and 1-grids to be the refined ones.

#### Closeness between entries

CLAG clusters elements in N according to E and to explain how it does it, we introduce the notion of *closeness* between pairs of entries. Let *S*_1_, *S*_2_ be two entries within the matrix such that *S*_1_ < *S*_2_. We say that *S*_1_ and *S*_2_ are *close* in a grid if they belong to the same interval in either the 0- or the 1-grid, or if they belong to two consecutive intervals, that is $I_{i}^{0},I_{i+1}^{0}$ or $I_{i}^{1},I_{i+1}^{1}$, and *S*_2_ belongs to *Δ*-*quantile*(S_1_).

Notice that for distributions of scores that are not heterogeneous, the definition of closeness can be greatly simplified: two entries *S*_1_,*S*_2_, with *S*_1_ < *S*_2_, are close if *S*_2_ belongs to the *Δ*-*quantile*(S_1_). For distributions which are possibly heterogeneous, the notion of grid turns out to be crucial but it should be observed that the concept of closeness could be stated by using the 0-grid only. The usage of the second grid (that is, 1-grid) is redundant here.

#### Environmental score

For a pair of elements V,Z∈N, we evaluate the closeness of the entries *A*(*V*,*X*) and *A*(*Z*,*X*) for each X∈E. We define the *environmental scoresS*_*env*_ by counting the number of characters *X* for which *A*(*V*,*X*) and *A*(*Z*,*X*) are close, say *K*, and we set $S_{\mathrm{env}}(V,Z)=\frac{K}{M}$ (for binary matrices, we count the number of *X* where *A*(*V*,*X*) = *A*(*Z*,*X*)). The set of characters *X* which are not close is denoted *Diff *(V, Z). For convenience, we renormalize the environmental scores *S*_*env*_ to the interval [−1,1]. A high environmental score reflects the fact that *V*,*Z* behave in a highly similar manner for all characters in E (Figure 1B), while a low score indicates a very different behavior within E (Figure 1C).

#### Clusters and affine clusters

To define a cluster in a matrix, we fix an element V∈N as a *cluster’s generator*, for a fixed *Δ*. For each pair of elements V,Z∈N, the cluster containing *V*,*Z* and generated by *V * is the largest set of elements W∈N such that the two following conditions are satisfied:

a. *S*_*env*_(*V*,*Z*) = *S*_*env*_(*V*,*W*),

b. *Diff*(V,Z) = *Diff*(V,W).

If no such *W * exist, the cluster is formed by the pair *V*,*Z*.

From the definition, it follows that a cluster is a subset of elements in N that behave similarly with respect to E. It also follows that two clusters generated by the same element might share at most one element, that is the generator. Clusters sharing several residues are generated by different elements. Notice that for a cluster *C*, the value *S*_*env*_(*V*,*Z*) is the same for all *Z* ∈ *C* (from a). We call this unique value *S*_*env*_(*C*). An *affine cluster* is a cluster *C* where *S*_*env*_(*C*) > 0, that is a cluster whose elements display identical scores (with respect to *Δ*) with at least a half of the environment. Strictly speaking, cluster affinity could be defined in more general terms with respect to a hyperparameter *δ*, by setting *S*_*env*_(*C*) > *δ*.

By increasing *Δ*, one expects clusters to get larger (since the number of pairs of entries that turn out to be equal up to *Δ* increases, and therefore the number of data points that are close increases) and possibly new ones to be created. This parameter renders the system flexible to clustering analysis and adaptable to multiple applications, the idea being that clusters detected by small *Δ*’s are the most meaningful and that significativity of clusters would decrease by enlarging *Δ*.

### Matrices with N⊆E

If N⊆E, then one can define an additional score, called the *symmetric scoreS*_*sym*_ of pairs of elements V,Z∈N, that establishes when *A*(*V*,*Z*) and *A*(*Z*,*V*) are identical up to *Δ* and where they are located along the distribution of entries.

#### Symmetric score

In order to evaluate the symmetric score of a pair V,Z∈N, we consider *A*(*V*,*Z*) and *A*(*Z*,*V*) and check for their closeness. *S*_*sym*_ is defined for close entries only, and for all other pairs is undefined. With no loss of generality, *A*(*V*,*Z*) < *A*(*Z*,*V*).

The definition of symmetric score for two close entries *A*(*V*,*Z*) and *A*(*Z*,*V*) is given by cases:

1. If *A*(*V*,*Z*) and *A*(*Z*,*V*) belong to $I_{n}^{0}$, we set *S*_*sym*_(*V*,*Z*) = *S*_*sym*_(*Z*,*V*) = 2·*n*.

2. If *A*(*V*,*Z*) and *A*(*Z*,*V*) belong to $I_{n}^{1}$, we set *S*_*sym*_(*V*,*Z*) = *S*_*sym*_(*Z*,*V*) = 2·*n* + 1.

3. If *A*(*V*,*Z*) and *A*(*Z*,*V*) belong to the two consecutive intervals $I_{n}^{0},I_{n+1}^{0}$ and to $I_{m}^{1},I_{m+1}^{1}$, and *A*(*Z*,*V*) is in *Δ*-*quantile*(A(V, Z)), then we set

(1) $$S_{\mathrm{sym}}(V,Z)=S_{\mathrm{sym}}(Z,V)=\left\{\begin{array}{c} 2\cdot n\;\;\;\text{if}\;I_{m}^{1}\leq I_{n}^{0}\\ 2\cdot m+1\;\;\text{if}\;I_{n}^{0}<I_{m}^{1} \end{array}\right)$$

where *I* < *J* means that the interval *I* starts before the interval *J*.

4. If *A*(*V*,*Z*) and *A*(*Z*,*V*) belong either to $I_{n}^{0},I_{n+1}^{0}$ or to $I_{m}^{1},I_{m+1}^{1}$, and *A*(*Z*,*V*) is in *Δ*-*quantile*(A(V, Z)), then we set *S*_*sym*_(*V*,*Z*) = *S*_*sym*_(*Z*,*V*) = 2·*n* or 2·*m* + 1 respectively.

The symmetric score of a pair of elements *V*,*Z* describes the approximate position of the *Δ*-quantile containing both *A*(*V*,*Z*) and *A*(*Z*,*V*) values in the distribution of entries. This mapping could be stated in different manners and we have chosen to do it with the help of two grids instead of one to obtain a more precise score function. For convenience, we renormalize the symmetric scores to the interval [−1,1].

#### Clusters and affine clusters taking into account symmetricity

We fix an element *V * as a *cluster’s generator*, for a fixed *Δ*. For a pair of elements V,Z∈N, the cluster containing *V*,*Z* and generated by *V * is the largest set of elements W∈N such that the three following conditions are satisfied:

a. *S*_*sym*_(*V*,*Z*) = *S*_*sym*_(*V*,*W*),

b. *S*_*env*_(*V*,*Z*) = *S*_*env*_(*V*,*W*),

c. *Diff*(V,Z) = *Diff*(V,W).

If no such *W * exist, the cluster is formed by the pair *V*,*Z*.

For a cluster *C*, there are unique values *S*_*env*_(*C*) and *S*_*sym*_(*C*). If *S*_*env*_(*C*) > 0 and *S*_*sym*_(*C*) > 0 then the cluster is affine. The symmetricity condition (a) imposes an extra requirement for similarity by enforcing elements in a cluster to behave symmetrically one to the other. The identification of such clusters might be useful in certain applications as illustrated for the dataset of residues in proteins discussed below.

### CLAG algorithm: the clustering step

**Figure 2:**
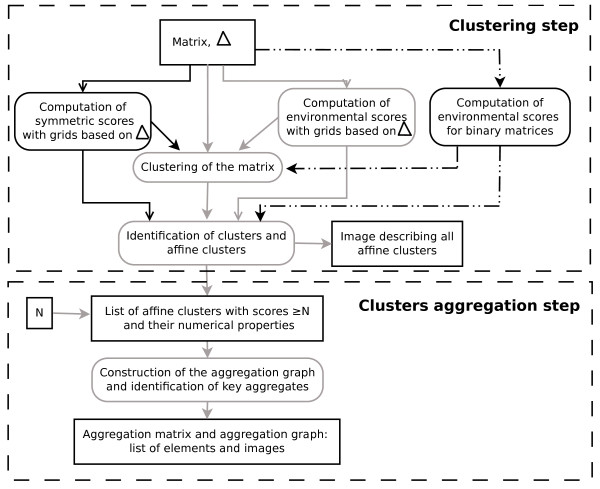
**CLAG flowchart.** Illustration of the different steps of the clustering method. The algorithm’s inputs are the matrix, *Δ* and the scores threshold *N* (*N* = 0 by default). The environmental score is computed differently for real-valued matrices (grey solid line) and binary matrices (dashed line). For matrices with N⊂E, CLAG computes the symmetric score too (black solid line).

CLAG is structured along two steps: a clustering step and a cluster aggregation step (Figure 2). The clustering step takes as input a matrix *A* and a value *Δ*, and goes as follows:

1. it computes environmental scores for all pairs of elements in N (symmetric scores are computed for matrices where N⊆E). Scores are normalized.

2. it clusters *A* by following conditions ab (abc, when *A* is such that N⊆E) as described above.

3. it identifies clusters and affine clusters.

4. it outputs a list of ranked affine clusters with respect to their environmental (and symmetric) scores and other numerical properties, and pdf images of the clustered matrix.

Notice that the input matrix is automatically renormalized to [0,1], if the matrix values do not belong to [0,1] already. The advantage of using renormalized values, is that they allow the user to visualize affine clusters with the R script developed for this purpose. Also, notice that when N⊆E, the algorithm can be executed in two manners: either by allowing the symmetricity condition to hold or not. When the condition is ignored, similarity will be computed for elements V,Z∈N such that |*A*(*V*,*Z*) − *A*(*Z*,*V*)| ≥ *Δ*.

Highest is the environmental score, closer is the behavior of the elements grouped in a cluster (with respect to the environment). This information is helpful to understand the structure of the set N and it could be used to identify elements that group together and those that are shared by several clusters while varying an environmental score threshold.

Notice that in the clustering step, the algorithm identifies the set of clusters generated by all elements of N and that there is no selection on generators that might bias clusters identification.

#### *The cluster aggregation step*

Clusters might share common elements and we wish to derive non overlapping sets of elements while keeping track of elements proximity. We do so for affine clusters and, possibly, for clusters with scores greater than a fixed positive threshold. We iteratively aggregate clusters in a graph as follows:

1. for any *n* clusters in the list, say *C*_1_,*C*_2_…*C*_*n*_, having the same (symmetric score, if it exists, and) environmental score, iteratively fuse together those clusters that share a common element and associate to the resulting cluster the same (symmetric score and) environmental score. Apply this step until no more clusters can be fused together. Rank the list of resulting clusters with the (highest symmetric score if it exists, and secondly, the) highest environmental score.

2. remove two clusters *C*_1_, *C*_2_ from the top of the ranked list; if *C*_1_,*C*_2_ share an element, then construct a graph whose labelled nodes are the elements of *C*_1_,*C*_2_ and whose edges are defined between all elements of *C*_1_, and between all elements of *C*_2_; we color the nodes of the graph with a unique color and call the resulting graph *an aggregate* . If *C*_1_,*C*_2_ do not have any element in common, construct a clique associated to each cluster and color them differently; the two labelled cliques are aggregates.

3. remove the first cluster *C* on the top of the list and check whether it shares some elements with existing aggregates. If it does, and the aggregates are *A*_1_…*A*_*k*_, where possibly *k* = 1, then construct an aggregate by adding to the *A*_*i*_’s the “new” nodes of *C* (that is, the nodes of *C* that do not already belong to the *A*_*i*_’s) and all edges between all nodes in *C*; if the shared nodes are several and colored differently, then color the new nodes of *C* with a new color. Otherwise, color the new nodes of *C* with the same color as the one used in *A*_*i*_. If *C* does not share any node with existing aggregates, then construct a clique and color it with a new color. The new graph forms an aggregate. Re-iterate until all clusters from the list are considered.

The resulting graph is called *aggregation graph*. Aggregates are disjoint graphs containing all nodes within clusters. We call *key aggregates* those subgraphs of the aggregation graph whose nodes are colored with the same color. Key aggregates describe clustering units that should be biologically interpreted.

In the following, without loss of generality, the term “key aggregate” will also be used to refer to the set of elements labeling the nodes of the key aggregate subgraph. Using sets, we present a toy example to illustrate the aggregation step. Let *C*_1_ = {1,2,3},*C*_2_ = {3,4,5}, *C*_3_ = {6,7,8},*C*_4_ = {8,9,10}, *C*_5_ = {5,10,11,12} be five affine clusters issued from the first step of the algorithm. Let *s*_1_,*s*_1_,*s*_2_, *s*_3_,*s*_4_ be their respective decreasing scores. By step 1, *C*_1_ and *C*_2_ are fused together in a set *C*_1,2_ = {1,2,3,4,5} because they have the same score and they share a common element. The set *C*_1,2_ has score *s*_1_. In step 2, the algorithm selects *C*_1,2_ and *C*_3_, that is the two clusters with highest score, it verifies that they share no common element and it labels *C*_1,2_,*C*_3_ with two different colors. Then, it selects *C*_4_ (in step 3), since it has the highest score among those clusters not yet examined. Cluster *C*_4_ shares an element with *C*_3_ and it is fused with *C*_3_ into a new set *C*_3,4_, keeping the color label of *C*_3_. By iterating step 3, *C*_5_ is considered. It shares an element with *C*_1,2_ and one with *C*_3,4_. The new set *C*_6_ = {11,12} is constructed by subtracting *C*_1,2_∪*C*_3,4_ from *C*_5_ and it is labelled by a new color. The three sets *C*_1,2_, *C*_3,4_ and *C*_6_ are the resulting key aggregates. Strictly speaking, the algorithm provides a colored graph structure that traces the relations between the different key aggregates (Additional file 1: Table S15).

It might be useful to rank aggregates with respect to the strength of the clusters that form them. This can be done by associating to an aggregate two *S*_*env*_ (*S*_*sym*_) scores: the first is the *S*_*env*_ (*S*_*sym*_) score of the first cluster entering the aggregate and the second is the *S*_*env*_ (*S*_*sym*_) score of the last cluster entering the aggregate.

#### Algorithmic complexity

The construction of the *N* × *N* matrix of environmental scores in the clustering step is realized in $O(N^{2}M)$. The sorting of the clusters generated by the clustering step is done in $O(N^{2}\log N)$ and the construction of the key aggregate sets in $O(N^{2})$. CLAG time performance is reported in Additional file 1: Table S1 for the biological datasets discussed later.

### Application to biological data

We analyze four datasets [17-19] to illustrate CLAG performance and large applicability. CLAG will be compared to *k*-means [1], c-means [15,16], MCLUST [9,10], hierarchical clustering [20,21] and Soft-Constraints Affinity Propagation (SCAP) [3] methods.

#### Breast tumor miRNA expression data

**Figure 3:**
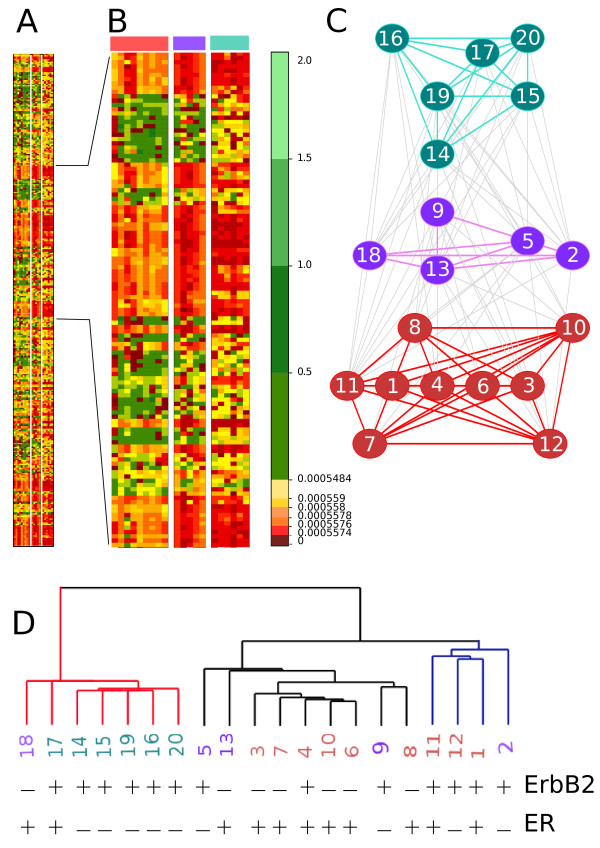
**Application on breast tumor samples data.** A panel of 20 different breast cancer samples [17]. **A**: matrix of key aggregates computed with CLAG, with *Δ* = 0.2 and *S*_*env*_(*A*) > 0, and zoom on the matrix. The red color scale is associated to small values and the green color scale to high values. The vast majority of values in the matrix is low and CLAG allows to distinguish them because of quantile segmentation. **B**: zoom on the matrix in A where the three aggregation graphs in C are indicated. **C**: aggregation graph produced by CLAG where three main clusters (produced by the first step of the algorithm and colored red, green and violet) are connected among each other by grey edges. Notice that the three clusters are indicated on the top of the zoomed matrix in B. Numbers labelling the nodes of the graph correspond to samples, that is columns in the matrix. **D**: dendrogram produced from the data clustered in A with a hierarchical clustering algorithm based closely on the average-linkage method of Sokal and Michener and developed in [21]. Three main clusters are found. The numbers are colored as in C and they are associated to columns in the matrix in B. For each sample, we denote the presence (+) or absence (-) of factors ErbB2 and ER whose overexpression is known to vary across cancer types.

A panel of 20 different breast cancer samples was chosen to represent three common phenotypes and was blindly analyzed for miRNA expression levels by microarray profiling [17]. For each breast cancer sample, 377 different miRNAs were considered. Hierarchical clustering (developed in [21] and based closely on the average-linkage method in [20]) generates a distance tree associating three known phenotypes of breast cancer (Figure 3D).

**Figure 4:**
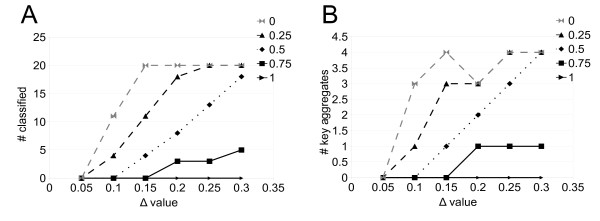
**CLAG on breast cancer data: clustering analysis.** Curves counting classified elements (**A**) and key aggregates **(B)** for increasing *Δ* values, are plot for different score thresholds.

When CLAG is applied to the dataset, it classifies all patients at *Δ* ≥ 0.15, for scores ≥0 (Figure 4A). For these thresholds, the number of key aggregates remains stable (Figure 4B) and we have chosen to describe in detail CLAG’s results for *Δ* = 0.20, where it detects three key aggregates. Its aggregation graph (Figure 3C) provides information on the proximity of the samples that is not described by hierarchical clustering (Figure 3D). Namely, there are two key aggregates, red and green in Figure 3C, that are formed by samples having a highly different behavior: we observe an almost complete absence of edges between the two key aggregates in the aggregation graph. The third key aggregate (violet in Figure 3C) plays a connecting role for the first two, with all its nodes that are linked to both green and red nodes. This division is well supported by the clinical interpretation of the samples. In fact, CLAG’s aggregates match well with three clinical pathologic features (that is the overexpression of the ErbB2, of the ER or of both receptors) that have been observed in gene expression profiling of clinically distinct breast cancer phenotypes: the green aggregate in Figure 3C corresponds to ErbB2 overexpression (6/6) and the red one corresponds to ER overexpression (8/9). The violet key aggregate presents less sharp tendencies with a presence of ErbB2 on 3/5 data points and of ER on 2/5 data points. The exact contingency table test for CLAG’s clustering (describing the three cancer phenotypes with respect to the three aggregates) gives *p* = 5.5*e*^−4^ and a sum of the probabilities of unusual tables of 0.025. These probabilities improve the ones computed over the tree organization in Figure 3D (describing the three cancer phenotypes with respect to the three main subtrees) giving *p* = 1.1*e*^−3^ and a sum of probabilities of unusual tables of 0.066. In both cases, the probabilities of unusual tables are small enough to reject the null hypothesis. (See Additional file 1: Table S4 for contingency tables and expected tables).

On this dataset, k-means, c-means and MCLUST fail clustering by proposing one or several clusters of single elements (see Additional file 1: Table S3 and Additional file 2).

#### Brain cancer gene expression data

The expression levels of more than 7000 genes for 42 patients have been monitored and classified in 5 different brain cancer diagnosis by an *a posteriori* assessment method [18] (10 medulloblastoma, 10 malignant glioma, 10 atypical teratoid/rhabdoid tumors, 4 normal cerebella, 8 primitive neuroectodermal tumors - PNET). To test CLAG classification we used a normalized dataset of 6010 genes where data arrays for each patient were filtered, log-normalized to mean zero and variance one [3]. We checked the outcomes against the assessment.

**Figure 5:**
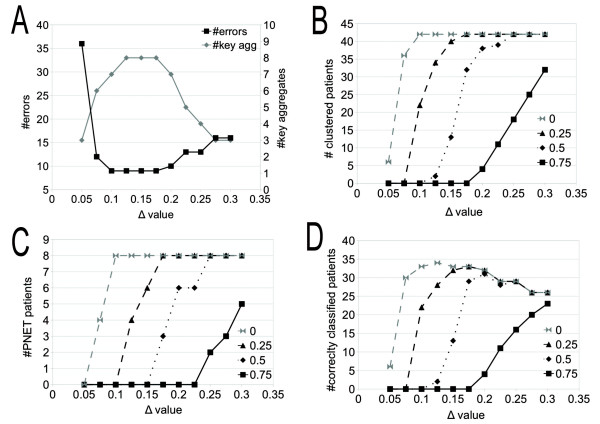
**CLAG on brain cancer gene expression data: error analysis.** Error analysis of CLAG clustering for gene expression data on brain cancer [18]. Data points are organized in five different pathologies and come from 42 patients. **A:** count of errors and key aggregates at increasing *Δ* values, computed on all affine clusters (that is, with scores ≥ 0). Errors count both misclassified and unclustered patients. Notice that for all points plotted at *Δ* ≥ 0.1, the number of clustered patients is maximal, that is 42 (see B). **B:** number of clustered patients evaluated on aggregation of clusters having scores greater than a fixed threshold. **C:** number of PNET patients aggregated at increasing *Δ* values, for different thresholds. Curves show that PNET patients aggregate slowly since they belong to clusters with low environmental and symmetric scores. **D:** number of patients that are correctly classified together.

For *Δ* ≥ 0.1 and by considering all affine clusters with scores  ≥ 0, CLAG aggregates all 42 patients (Figure 5B). For *Δ* = 0.1, CLAG produces 7 key aggregates with 9 errors (Figure 5A), where errors count both misclassified patients and unclassified patients. Several isolated clusters of the same diagnosis are found. The medulloblastoma patients are all grouped together. Normal patients form a separate group and do not mix. Errors are mostly due to misclassification of PNET patients that mix with glioma and medulloblastoma patients.

While *Δ* increases until 0.2, the number of correctly classified patients remains essentially stable and the number of key aggregates, after augmenting for a while, gets smaller (Figure 5A). As expected, aggregation of clusters with increasingly large *Δ* values, shows an increased number of errors for a decreased number of key aggregates. See Figure 5ABD.

With *Δ* = 0.1 and score threshold = 0.25, clustered patients decrease to 22 and they organize in 6 diagnosis specific key aggregates, with no mix. No PNET patients are classified. This suggests that clusters obtained for scores ≥0 are formed by a core of patients that are well classified and that misclassified patterns, like PNET, are peripherical cluster elements (Figure 5C). The possibility to provide information on the structure of the dataset and on the internal organization of the clusters is a feature of CLAG.

In [18], patients were clustered using a hierarchical clustering. Even though the structure of the clustering is similar to the one we obtained, there is no clear-cut partition in 5 groups of patients, several diagnosis mix together and PNET patients appear in several distinguished subtrees. Our results have been also compared to the ones obtained with SCAP [3]. SCAP outputs 4 clusters with 8 errors. Normal patients form a separate group and all SCAP errors are due to misclassification of PNET patients that are found spread on three distinguished clusters associated to malignant tumor diagnosis. Both CLAG and SCAP provide information on the structure of the dataset. k-means, c-means and MCLUST propose clusters that highly mix the five diagnosis. See Additional file 1: Table S2 and Additional file 3 for a comparative assessment.

#### Coevolved residues in protein families data

A large number of coevolution analysis methods investigate evolutionary constraints in protein families via correlated distribution of amino-acids in sequences. Given a protein family, they produce a square matrix of coevolving scores between pairs of alignment positions in the sequence alignment associated to the protein family [19,22-25]. Clustering of the score matrix helps to identify groups of coevolving residues often characterizing important functional and structural properties for the protein family. The identification of groups displaying the highest signals of coevolution has been previously realized by hand.

**Figure 6:**
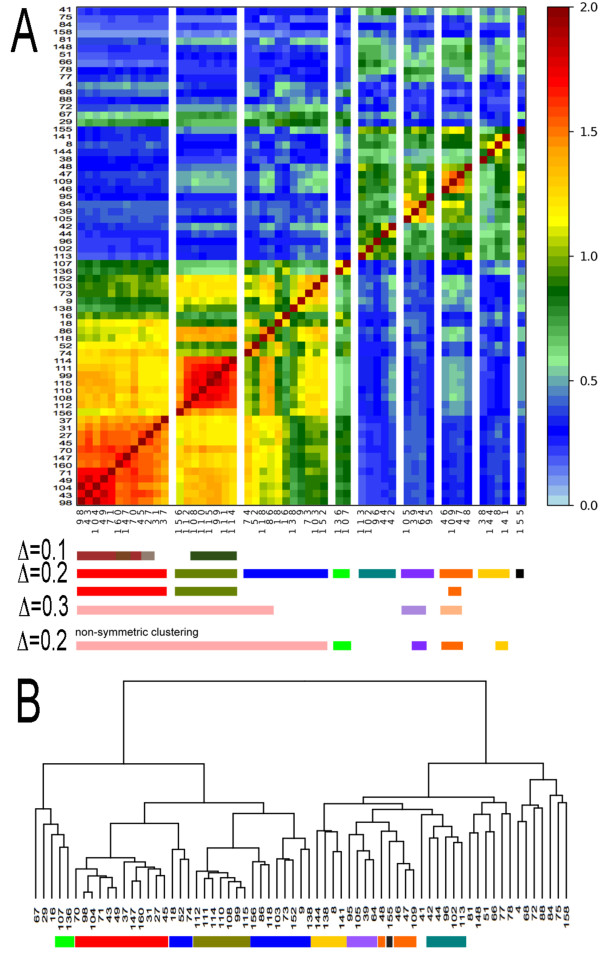
**CLAG on a coevolution scores matrix.** Clustering of the MST matrix of coevolution scores for the globin protein family [19]. It is a squared matrix on 67 alignment positions selected by the MST method as susceptible to coevolve. **A**: Slices of the clusterized matrix associated to all key aggregates obtained with *Δ* = 0.2, and environmental and symmetric scores ≥ 0.5. The order of the 67 positions in the y-axis follows the order of the key aggregates positions in the *x*-axis (from left to right). Positions belonging to key aggregates obtained for *Δ* = 0.1,0.2,0.3 with environmental and symmetric scores = 1 are reported at the bottom of the matrix with the help of colored bars. For *Δ* = 0.1,0.3 and *Δ* = 0.2 (bottom), the score of aggregation is = 1. For *Δ* = 0.2 (top), scores are ≥ 0.5. Key aggregates obtained without considering the symmetricity condition in CLAG are reported (bottom) for *Δ* = 0.2 and environmental scores = 1. **B**: hierarchical clustering of the dataset where key aggregates of *Δ* = 0.2 and scores ≥ 0.5 detected in A are highlighted (colors as in A).

**Figure 7:**
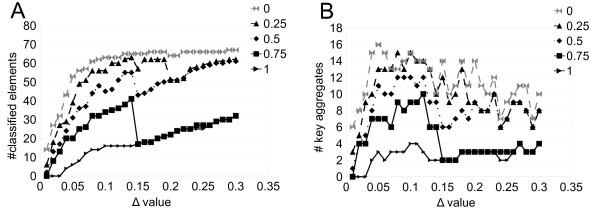
**CLAG on the globin dataset: clustering analysis.** Curves counting classified elements **(A)** and key aggregates **(B)** for increasing *Δ* values, are plot for different score thresholds.

We applied CLAG to the coevolution score matrix produced by the coevolution analysis method MST [19] on the globin protein family, to automatically detect coevolving groups of residues. By increasing *Δ*, CLAG detects clusters of maximal scores with progressively larger sizes as well as new clusters (Figures 6A and 7AB). There are two main key aggregates that are detected at *Δ* = 0.1 and grow larger at *Δ* = 0.2. (At *Δ* = 0.3, they begin to collapse.) The first key aggregate (red) corresponds to the conserved binding site enveloping the haem and the second key aggregate (olive green) corresponds to a group of residues that is known to be associated to the allosteric function [19,26]. By analyzing clusters with weaker strength, 7 more key aggregates were found and three of them (orange, yellow and violet) have been highlighted in [19] too, as belonging to the globin subunits binding sites. These five key aggregates are the first ones to form during the aggregation step (Additional file 1: Table S6).

Notice that for this dataset, N⊆E and that the aggregates were found by applying CLAG under the symmetricity condition. The identification of clusters grouping elements with symmetric behavior turned out to be important for distinguishing the red and the olive green aggregates, known to present functionally distinct roles for the globin (see Figure 6A and Additional file 1: Figure S3). Without symmetricity, the red aggregate would be identified at *Δ* = 0.1 (Additional file 1: Figure S3) but the olive green would systematically collapse with other aggregates. In general, one can observe that with no symmetricity condition, aggregates will be larger, possibly characterized as the join union of aggregates determined with symmetricity, and possibly including other residues, that were not considered as coevolving by the symmetric condition.

Agglomerative hierarchical clustering [26,27] detects the red and olive green clusters but it also detects other clusters as subtrees of comparable height without distinguishing them (Figure 6B).

When we compare *k*-means to CLAG on this dataset, we observe that several key aggregates detected by CLAG are grouped within a single *k*-means cluster (Additional file 4). In particular, red and green key aggregates are grouped together (Additional file 1: Table S5) and this hints that no biological interpretation can be associated with *k*-means clusters. Slightly better results are obtained with c-means, MCLUST and SCAP, where the overall clusters structure is similar to the one found by CLAG, but no clear cut identification of our two stronger key aggregates is obtained (Additional file 4). Our red and olive green key aggregates are separated in distinguished clusters but mixed with many data points mainly belonging to the blue key aggregate. Also, for SCAP, convergence into 8 clusters classifying all 67 alignment positions is obtained for *p* = 0.13, in less than 100 iteration steps (Additional file 1: Table S5 and Additional file 1: Figure S4), but SCAP greatest stability in *p* variation is reached for 2 clusters (Additional file 1: Figure S5), corresponding to the two large subtrees of the hierarchical tree in Figure 6B. Notice that all residues are considered by these clustering tools and that many of them do not coevolve.

#### CLAG in synthetic datasets

**Figure 8:**
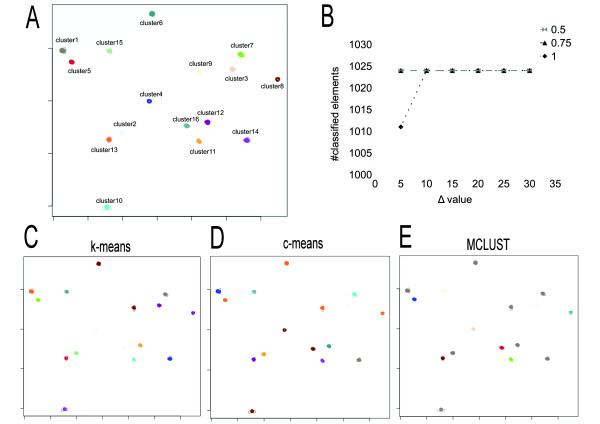
**CLAG on the 128-dimensional synthetic dataset.A:** the 128-dimensional dataset contains 1024 points and 16 clusters generated with a gaussian distribution (http://cs.joensuu.fi/sipu/datasets/DIM128.txt). CLAG perfectly distinguished the 16 clusters when run with *Δ* = 0.05 and scores ≥ 0.5. **B:** curves associated to different score thresholds describing the number of elements that are clustered by CLAG while varying *Δ* values. Note that the number of elements is 1011 for *Δ* = 0.05 and maximal scores. Different clustering algorithms were run on this dataset: k-means **(C)**, c-means **(D)**, MCLUST **(E)**. k-means and c-means were run with 16 clusters, and MCLUST with “ellipsoidal, equal variance with 9 components” as best model (note the 8 grey clusters). For *k*-means, clusters 1, 13 are split in several *k*-means clusters while clusters 3, 8 (violet) and 4, 16 (light blue) are fused together. *c*-means clusters the original ensemble in only 11 clusters: clusters 10, 4, 16 (brown) and 5, 6, 8, 9 (orange) are grouped together. In A, C, D, E elements are represented by circles. Different clusters are distinguished by different colors. Figures ACDE are realized by plotting the first two columns of the matrix describing the dataset.

We run CLAG on six different synthetic datasets with Gaussian clusters, each of them constituted by 1024 vectors, organized in 16 clusters and defined in 32, 64, 128, 256, 512 and 1024 dimensions respectively. CLAG succeeds in clustering correctly all datasets for *Δ* ≥ 0.1 (Figure 8, Additional file 1: Figures S6-S10 and Additional file 5) by producing 16 key aggregates describing the 16 original clusters. *k*-means provides misclassification errors on all datasets while *c*-means behaves well on dimensions 32 and 64, and optimizes to less than 16 clusters datasets of higher dimension. MCLUST clusterizes based on ellipsoidal models with a very small number of components and, in this manner, it fuses together several clusters, for all the multi-dimensional datasets. In dimension 1024, it generates a single huge cluster. In conclusion, as the dimension of the data goes higher all methods produce classification errors whereas CLAG continues to identify correctly the 16 clusters.

Also, we generated 2D sets of points with different shapes and degrees of density and checked the performance of k-means, c-means, MCLUST and CLAG on these datasets (Additional file 6). When sets of points are well separated in space, CLAG clusters them properly independently of shapes (Additional file 1: Figures S12-S14B). The same happens for c-means but neither for k-means nor MCLUST. When sets of points overlap together, CLAG tends to cluster the sets in a single key aggregate (Additional file 1: Figures S13 and S14) contrary to the other tools that prefer to split the datasets in small clusters, sometimes erroneously (see Additional file 1: Figure S14BCDE for the blue shape in Additional file 1: Figure S14A).

#### CLAG’s parameterization

CLAG is based on two parameters, *Δ* and the threshold for environmental and symmetric scores. These parameters can be used together for evaluating whether all elements of the dataset are supposed to be clustered together or for determining how many clusters a dataset is made of. The main point is to find an interval of values *Δ* where the number of clustered elements as well as the number of key aggregates remains essentially stable. For the brain cancer dataset (Figure 4), stability is observable for the curve dependent on scores threshold = 0 starting from *Δ* ≥ 0.15; for the breast cancer dataset, score threshold = 0 and *Δ* ≥ 0.1 identify the clustering of all data points (Figure 5AB); the globin dataset displays a stable behavior for the curve associated to threshold = 1 and for *Δ* values > 0.175 (Figure 7).In the case of the synthetic datasets, stability is reached for *Δ* ≥ 0.10 and arbitrary thresholds (Figure 8). It could be envisageable to implement these criteria to render automatic the identification of best parameter values.

## Conclusions

CLAG is an unsupervised non-hierarchical and deterministic clustering algorithm applicable to *M* × *N* matrices. Its range of application is spread as illustrated by the datasets we discussed. Contrary to algorithms like the *k*-means, the number of clusters to identify is not specified, but there is a parameter to the algorithm, *Δ*, which influences the number of clusters that can be predicted. This parameter modulates the proximity between elements that are accepted to be “similar” or “close”. Based on *Δ*, the clustering step determines if two elements in N are similar with respect to the environment E (and, possibly, if they are symmetric) providing, in this way, a numerical score that describes the strength of the signal. The aggregation step combines clusters sharing the same data points and it produces key aggregates, that is disjoint clusters. Clustering depends on *Δ* and aggregation depends on the scores threshold.

An important feature is that CLAG does not try to clusterize all data points, but it combines just those that are sufficiently similar to be clustered together. Because of this relaxed clustering constraint, after the clustering step, the user learns which data points drove the clustering with respect to *Δ*. The gradual extension and creation of clusters with increasing *Δ* values, provides the user with information on the structure of the dataset.

The cluster structure present in biological datasets can be systematically investigated with CLAG. This underlying structure between data points is typically not a tree but a graph, and CLAG provides an aggregation graph describing it.

Known clustering methods ask for a data point to belong to at most one cluster. For certain applications, this is a limitation. For instance, for coevolution score matrices, a fixed alignment position in a protein family could be subjected to more than one evolutionary constraint and therefore might play several roles for the protein. Unlike other approaches, CLAG allows for a position to belong to several clusters. Hence, the user can extract useful information from the clustering step and eventually use the outcomes of this step as a clustering result.

For the user, scores are relevant to evaluate clusters strength and to decide whether clusters should be considered important or not for their analysis. This numerical feature is missing for the hierarchical clustering where it becomes hard, at times, to choose among subtrees based on their height. The globin analysis is an example of this (Figure 6AB). Also, scores reflect the structure of the dataset. They highlight where closest data points are located and which subsets collapse together if more relaxed proximity conditions, that is larger *Δ* values, are allowed.

CLAG second step (producing key aggregates) is applied only on affine clusters, that is clusters with positive environmental (and possibly symmetrical) score(s). Notice that the general notion of affinity, asking for *S*_*env*_(*C*) > *δ* for some hyperparameter *δ* that has been mentioned above could be used to parameterize further the algorithm to allow the user to set a threshold on affinity for the aggregation step. The definition of affine cluster, setting *δ* = 0, asks for at least half of the characteristics of a data point to be shared with the other data points of the cluster, and it seems to set a reasonable condition to compute key aggregates of sufficiently high strength.

We should warn potential users that the definitions of environmental score and affine cluster implicitly assume that all the *M* characters are equally important for clustering purposes. This can be a strong assumption, as in many situations it could not be known whether all the characters in a dataset are relevant for clustering purposes.

CLAG has been compared to various clustering approaches on four biological datasets, and showed to be more informative and accurate than hierarchical agglomerative clustering and *k*-means. The clustering of the dataset of coevolving residues showed SCAP to furnish inaccurate results due to its need to consider all data points instead of a subset of those. On the brain cancer dataset, where the full set of patients should be clustered, SCAP and CLAG showed a comparable performance. Finally, CLAG does not suffer of the convergence problem proper to AP and SCAP, and always leads to clustering. Compared to MCLUST, CLAG shows better performance in clustering multidimensional datasets where the size of the environment *M* is much larger than the size of the set of elements *N*. This is seen for brain cancer and breast cancer datasets as well as for multidimensional synthetic datasets. MCLUST outcomes are not unexpected. In fact, the Gaussian mixture models implemented in MCLUST may become over-parametrized and give poor performances on multidimensional datasets [28,29]. Similarly, the poor performances of MCLUST and of k-means on the synthetic 2D datasets is likely due to the fact that these two clustering methods implicitly assume that all groups of elements have spherical or elliptical shapes, which is not the case in the described examples. We should notice that on other datasets, as the IRIS dataset for instance, characterized by few dimensions and a large number of elements, CLAG does not perform well compared to the success of mixture-model-based methods [9,13,30] that detect the correct number of Iris flower groups by selecting variables appropriately, showing that clustering on all variables always provides an ambiguous result on this dataset.

## Methods

### Implementation

CLAG takes as input a matrix and a *Δ* value and it outputs a text file with a list of clusters together with scores and parameter values, a list of key aggregates, an aggregation graph, and clustered matrices. CLAG is written in perl, it uses the R-package [31] to draw matrices (http://www.r-project.org/) and it draws graphs with neato found in Graphviz [32] (http://www.graphviz.org/Credits.php). neato draws graphs only when they are not too large (about 100 nodes; notice that 100 corresponds to *N* and not to *M*; *M* can be much larger as in Figure 4), and for graph with more than 100 nodes, no pdf file is generated. The description of the aggregation graph is output on a text file. How to use it and examples are found in the Additional file 1.

CLAG is freely available under the GNU GPL for download at http://www.ihes.fr/∼carbone/data11. It is supported on Linux and Mac OSX. Sample datasets are given. Parameters and instructions are described in Additional file 1.

### Comparative tools and data

Hierarchical clustering, *k*-means, *c*-means and MCLUST were performed with functions in the R-package. Affinity Propagation (AP) was used online at http://www.psi.toronto.edu/affinitypropagation/webapp/ and, for all our datasets it did not converge. Soft-Constraint Affinity Propagation (SCAP), showed to improve AP performance [3] and was run on a distribution provided by the authors after request.

Six multi-dimensional synthetic datasets were downloaded from http://cs.joensuu.fi/sipu/datasets/. Three were generated with the software DataGenerator.jnlp [33], downloadable at http://webdocs.cs.ualberta.ca/∼yaling/Cluster/Php/index.php. The three generated datasets contain 500 points and 5 clusters at different density levels: G4 was generated with difficulty level=1 and density level= 3, and G5, G6 were generated with difficulty level=2 and density level= 3. The software ELKI [34] was used to represent classification results, for all methods, on synthetic datasets G4, G5, G6 in the Additional file 1.

The exact contingency table computation has been realized on the website http://www.physics.csbsju.edu/cgi-bin/stats/exact.

## Competing interests

The authors declare that they have no competing interests.

## Authors’ contributions

AC and LD designed the algorithm, selected and analyzed the four experiments illustrating the applicability and the performance of the algorithm. LD implemented the tool. Both authors read and approved the final manuscript.

## Supplementary Material

Additional file 1 CLAG instructions and Figures issued from the datasets analysis. A list of instructions for running CLAG and extra figures for the analysis of the four datasets discussed in the article are given. Click here for file

Additional file 2 CLAG executions on the breast cancer dataset. CLAG executions on the breast cancer dataset are detailed with respect to parameters variation. Executions of other clustering tools (k-means, c-means, MCLUST) are also reported. Click here for file

Additional file 3 CLAG executions on the brain cancer dataset. CLAG executions on the brain cancer dataset are detailed with respect to parameters variation. Executions of other clustering tools (k-means, c-means, MCLUST) are also reported. Click here for file

Additional file 4 CLAG executions on the globin dataset. CLAG executions on the globin dataset are detailed with respect to parameters variation. Executions of other clustering tools (k-means, c-means, MCLUST) are also reported. Click here for file

Additional file 5 CLAG executions on all multi-dimensional datasets and best models computed by MCLUST. CLAG executions on all multi-dimensional datasets are detailed with respect to parameters variation. BIC values for best model selection are reported for MCLUST. Click here for file

Additional file 6 CLAG executions on synthetic datasets G4, G5, G6. CLAG executions on synthetic datasets G4, G5, G6. Executions of other clustering tools (k-means, c-means, MCLUST) are also reported. Click here for file
