# Supplementary material for: CLAG: an unsupervised non hierarchical clustering algorithm handling biological data
Source: BMC Bioinformatics. 2012 Aug 8;13:194. doi: 10.1186/1471-2105-13-194 (PMC3519615; doi:10.1186/1471-2105-13-194)
Supplement: Additional file 1 — CLAG instructions and Figures issued from the datasets analysis. A list of instructions for running CLAG and extra figures for the analysis of the four datasets discussed in the article are given. [file 1471-2105-13-194-S1.pdf]

Supporting Information

1 Instructions for running CLAG

The program CLAG has been designed to cluster sets of multidimensional data by looking for correlated data within the set.

1. Instructions for running CLAG

CLAG has been designed to detect clusters in a $M \times N$ matrix, where N is the number of elements in a set $\mathcal{N}$ and M is the number of properties $\mathcal{E}$ describing the elements in $\mathcal{N}$. We call $\mathcal{E}$ the *environment*. CLAG is split into two steps:
a. the detection of clusters of elements
b. the aggregation of clusters obtained in step 1 into an aggregation graph and the visualization of key aggregates.

The first part of the algorithm needs as input the data of an $M \times N$ matrix loaded from a file called "input.txt", each line represents a pair of elements and the associated score (separated by a space), that is:

<element of N> <element of M> score

CLAG provides a list of files describing the clusters identified with respect to the parameter Δ.

The second part computes the key aggregates, constructs the aggregation graph and provides a set of figures representing clusters and key aggregates for each allowed value of the parameter Δ, parameterizing the analysis by quantiles of the score distribution.

To run the program on a general matrix using the command line, write:

./exe-RCommand.pl - f=/folder/ -p=n -k=i -d=X

where n takes 3 possible values depending on the input we have and the clustering we look for:
1 a general matrix
2 a binary matrix
3 a matrix where $\mathcal{N} \subset \mathcal{E}$

Parameters k and d are optional. Notice that i is the lower bound of environmental scores (and possibly symmetric scores) accepted in the aggregation analysis. If k is missing, then by default it takes value 0. Notice that i does not play a role in the clustering step where all affine clusters are identified (that is, $i = 0$). The value X can be any integer. If -d is not specified, then CLAG computes all values 5, 10, 20, 40 (standing for $\Delta = 0.05, 0.1, 0.2, 0.4$) by default. For binary matrices, the parameter d is useless.

2. CLAG output files

CLAG works on input matrices whose real values lie in the interval $[0, 1]$ (input.txt). If the input matrix does not satisfy this condition, CLAG renormalizes the values of the matrix and keeps the original matrix into the file inputOriginal.txt.

In the first step, CLAG outputs several files. They enumerate the list of all clusters and their associated scores. In case of general or matrices where $\mathcal{N} \subset \mathcal{E}$, the files associated to the clustering of the matrix at a given value Δ, say $\Delta = 0.M$, are:

- CLUSTERFILE-COMPLETE-M.txt: each line is a description of a different cluster C and their corresponding scores. Namely, it reports: the Δ value, the percentage of elements $Y \in \mathcal{E}$ such that $A(X,Y) = A(Z,Y)$

where $X, Z \in \mathcal{N}$ and X is the generator, the list of elements in $\mathcal{E}$ belonging to the set $Diff(X, Z)$ (if the list is empty, the value is -1), the list of elements in the cluster C, the environmental score $S_{env}(C)$. Each information is separated by a ":".

- CLUSTERFILE-M.txt : each line is a description of a different affine cluster and their corresponding scores. See above for each information reported.

In the second step, CLAG outputs files describing key aggregates:

- aggregation-M.txt : list of key aggregates elements, rank of the key aggregates (determined by the highest symmetric score, if it exists, and secondly by the highest environmental score), scores of the first and the last clusters merged by the algorithm. In the case of a general matrix, there will be the two environmental scores, and in the case of a matrix where $\mathcal{N} \subset \mathcal{E}$, the scores will be four, the two symmetric and the two environmental scores. Each information is separated by a ":".
- GRAPH-aggregation-M.dot : input for neato
- GRAPH-aggregation-M.pdf : output from neato.

Also, CLAG generates several figures describing the unclusterised matrix (Matrix.pdf), the aggregation graph generated by neato (a package of graphviz), the cluster aggregation matrix (Matrix-aggregated-M.pdf or Matrix-aggregated.pdf; this matrix displays all key aggregates constructed out of clusters with scores $\geq i$), the clustered matrix for all scores (Matrix-Clusterized-M.pdf or Matrix-Clusterized.pdf) and the clustered matrix for environmental scores = 1 (and symmetric score = 1 in the case of a special case matrix) (Matrix-Clusterized-M-Scores1.pdf or Matrix-Clusterized-Scores1.pdf). The generation of these two last matrices can be dropped off on a comment line in the code. This option has been imposed because, at times, the generated files are too large and the user might want to avoid their generation. All files generated by R and neato are in pdf format. Note that the files beginning with "PR-SCORING-output" contain the matrix given to R for the generation of the corresponding pdf files. The commands given to R are contained in the files beginning with "R_COMMAND".

In the case of a binary clustering, the first and the second step of the algorithm are independent of the parameter Δ and the unique files generated by CLAG are: CLUSTERFILE-COMPLETE.txt, CLUSTERFILE.txt, aggregation.pdf, GRAPH-aggregation.dot, GRAPH-aggregation.pdf.

CLAG draws graphs with neato found in Graphviz (Ellson J, Gansner ER, Koutsofios E, North SC, Woodhull G (2001) Graphviz - Open Source Graph Drawing Tools. *Symposium on Graph Drawing - GD* 483-484), downloadable at http://www.graphviz.org/Credits.php. Notice that neato draws graphs only when they are not too large (about 100 nodes; notice that 100 corresponds to N and not to M; M can be much larger as in Figure 1). For graph with a large number of nodes, there will be no ps file generated. Only file .dot will be output as well as the matrix of key aggregates.

CLAG requires perl, R, Graphviz. CLAG uses the R-package (R Development Core Team (2008) R: A language and environment for statistical computing. R Foundation for Statistical Computing, Vienna, Austria. ISBN 3-900051-07-0, URL http://www.R-project.org.) downloadable at http://www.r-project.org/.

2. CLAG global analysis

**Dataset**	**Size**	**CLAG step**	**Δ values**				
			**0.05**	**0.1**	**0.15**	**0.20**	**0.25**
Breast cancer	322×20	clustering	0.921s	0.822s	0.796s	0.745s	0.679s
		aggregation	0.014s	0.015s	0.019s	0.018s	0.020s
Brain cancer	6010×42	clustering	71.086s	55.902s	46.102s	39.565s	34.890s
		aggregation	0.0083s	0.035s	0.07s	0.097s	0.072s
Globine	67×67	clustering	12.210s	9.219s	8.088s	7.458s	6.805s
		aggregation	0.014s	0.024s	0.036s	0.035s	0.044s

SI Table 1. CLAG computation time. Time is computed for the first and second step of CLAG execution on the data set discussed in the article, for increasing values of the Δ parameter. For each Δ, aggregation is performed on affine clusters.

3. Brain cancer dataset analysis

k-means	
Cluster	Details
C1	31 32 33 34 41
C2	2 3 4 5 6 7 8 9 10 35 40
C3	1 18 21 22 23 24 25 26 27 28 29 36
C4	30 39
C5:	11 12 13 14 15 16 17 19 20 37 38 42
**c-means**	
C1	1 4 6 7 9 16 18 21 22 23 24 25 26 27 28 29 30 35 36 38 39
C2	2 3 5 8 10 11 12 13 14 15 17 19 20 31 32 33 34 37 40 41 42
**MCLUST**	
C1	1 2 3 4 5 6 7 8 9 10 35 40
C2	11 12 13 14 15 16 17 19 20 31 32 33 34 37 38 39 41 42
C3	18 21 22 23 24 25 26 27 28 29 30 36

SI Table 2. k-means, c-means and MCLUST classification on the brain cancer dataset. k-means and c-means have been run for 5 clusters. MCLUST selected the "VVI" (diagonal, varying volume, varying shape) with 3 components as best model. The BIC value could not be evaluated for all components and models.

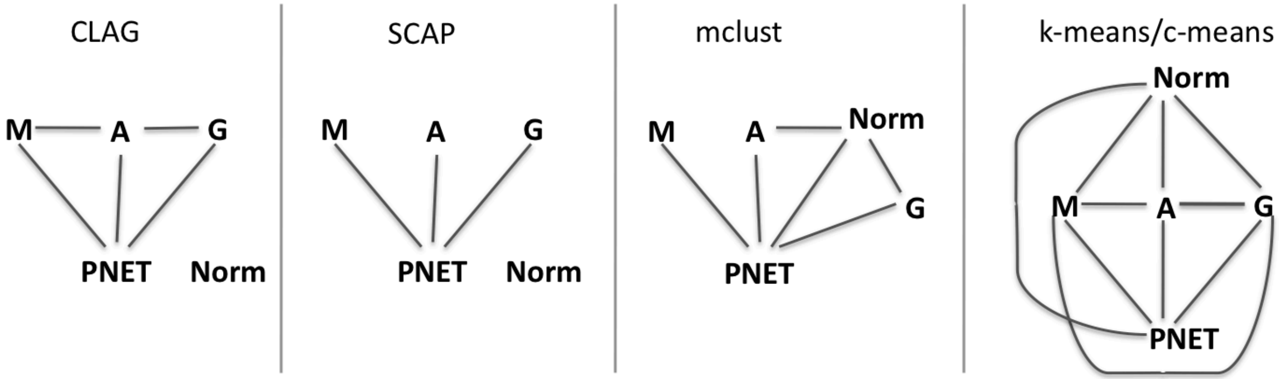

SI Figure 1. Comparison between different classification tools on brain cancer data. The brain cancer dataset is constituted by five sets: Medulloblastoma (M), malignant glioma (G), atypical teratoid/rhabdoid tumors (A), normal cerebella (Norm), primitive neuroectodermal tumors (PNET). Each graph is associated to a method and it represents the mix of the five sets after clustering. The five sets label the nodes of the graphs and each edge in the graph describes the coexistence of elements in the sets in at least one cluster. Clusters for k-means, c-means and MCLUST are reported in SI Table 2.

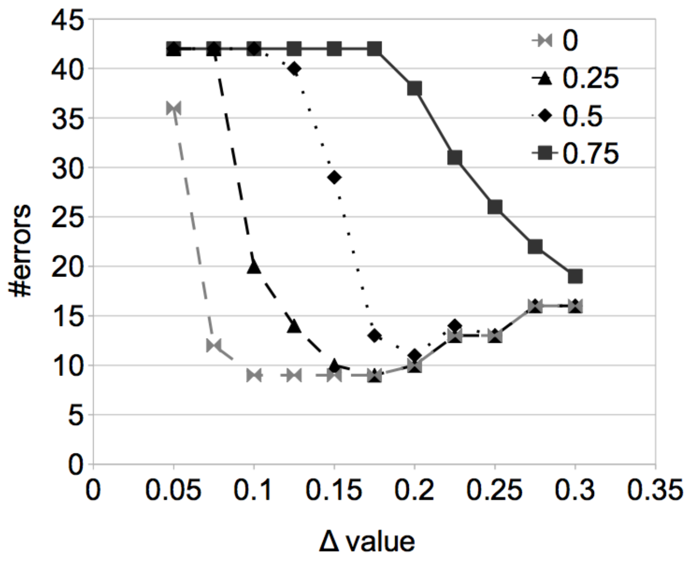

SI Figure 2. CLAG applied to brain cancer data: error analysis. Curves reporting the number of errors associated to aggregations based on clusters with scores satisfying a certain threshold. The curves describe how the errors decrease by varying Δ. Errors are misclassified and unclustered datapoints.

4. Breast cancer dataset analysis

**k-means**	
Cluster	Details
C1	3
C2	10
C3	1 2 4 5 6 7 8 9 11 12 13 14 15 16 17 18 19 20
**c-means**	
C1	3 9 10
C2	1 2 4 5 11 12 13 14 15 16 17 18 19 20
C3	6
**MCLUST**	
C1	1 2 4 5 11 14 15 16 17 18 19 20
C2	3
C3	6
C4	7
C5	8
C6	9
C7	10
C8	12
C9	13

SI Table 3. k-means, c-means and MCLUST classification on the breast cancer dataset. k-means and c-means have been run for 3 clusters. MCLUST selected ”EEI” (spherical, equal volume) with 9 components as best model. The BIC value could not be evaluated for all components and models.

	CLAG				Expected			
	ErbB2	ER	Both	Total	ErbB2	ER	Both	Total
green	5	0	1	6	2.70	2.10	1.20	6
red	1	5	3	9	4.05	3.15	1.80	9
blue	3	2	0	5	2.25	1.75	1.00	5
Total	9	7	4	20	9	7	4	20

	Hierarchical Clustering				Expected			
	ErbB2	ER	Both	Total	ErbB2	ER	Both	Total
red	5	1	1	7	3.15	2.45	1.40	6
black	2	6	1	9	4.05	3.15	1.80	9
blue	2	0	2	4	1.80	1.40	0.800	5
Total	9	7	4	20	9	7	4	20

SI Table 4. Contingency tables computed for CLAG and hierarchical clustering. Contingency tables and expected tables for CLAG (top) and hierarchical clustering (bottom) results are reported. The table associated to CLAG results represents the distribution of overexpression signals (ErbB2, ER and both) obtained within the three clusters (green, red and blue) highlighted in Figure 5D. The table associated to the hierarchical clustering results is organised around the three clusters (red, black and blue) corresponding to the three subtrees in Figure 5D.

5. Globin dataset analysis

**k-means**	
Cluster	Details
C1	9 18 27 31 37 43 45 49 52 70 71 73 74 86 98 99 103 104 108 110 111 112 114 115 118 147 152 156 160
C2	38 41 51 66 75 77 78 81 84 88 144 148 158
C3	8 39 42 44 46 47 48 64 95 96 102 105 109 113 141 155
C4	4 16 29 67 68 72 107 136 138
**c-means**	
C1	4 29 38 41 51 67 68 72 75 77 78 81 84 88 136 144 148 158
C2	8 39 42 44 46 47 48 64 66 95 96 102 105 109 113 141 155
C3	9 73 86 99 103 107 108 110 111 112 114 115 118 138 152 156
C4	16 18 27 31 37 43 45 49 52 70 71 74 98 104 147 160
**MCLUST**	
C1	4 68 72 75 77 84 88 158
C2	8 38 39 41 42 44 48 51 64 66 78 81 95 96 102 105 113 141 144 148 155
C3	9 73 86 103 118 138 152 156 16 29 67 107 136
C4	18 52 74 27 31 37 45 147 160
C5	43 49 70 71 98 104
C6	46 47 109
C7	99 108 110 111 112 114 115
**SCAP**	
C1	16 18 27 29 31 37 43 45 49 52 68 70 71 74 98 104 107 136 147 160
C2	86 99 108 110 111 112 114 115 118 156
C3	41 42 44 48 51 66 96 102 113
C4	9 67 72 73 88 103 138 152
C5	4 39 64 77 78 84 95 105
C6	46 47 109 155
C7	81 148 158
C8	8 38 75 141 144

SI Table 5. k-means, c-means and MCLUST classification on the globin dataset. k-means and c-means have been run for 4 clusters. MCLUST selected ”VII” (diagonal, varying volume and shape) with 9 components as best model. The best model occurs at the max # of components considered and the optimal number of clusters occurs at max choice. SCAP clusters were obtained with $p = 0.13$. Residues belonging to CLAG red cluster and to CLAG green cluster are highlighted in red and green respectively.

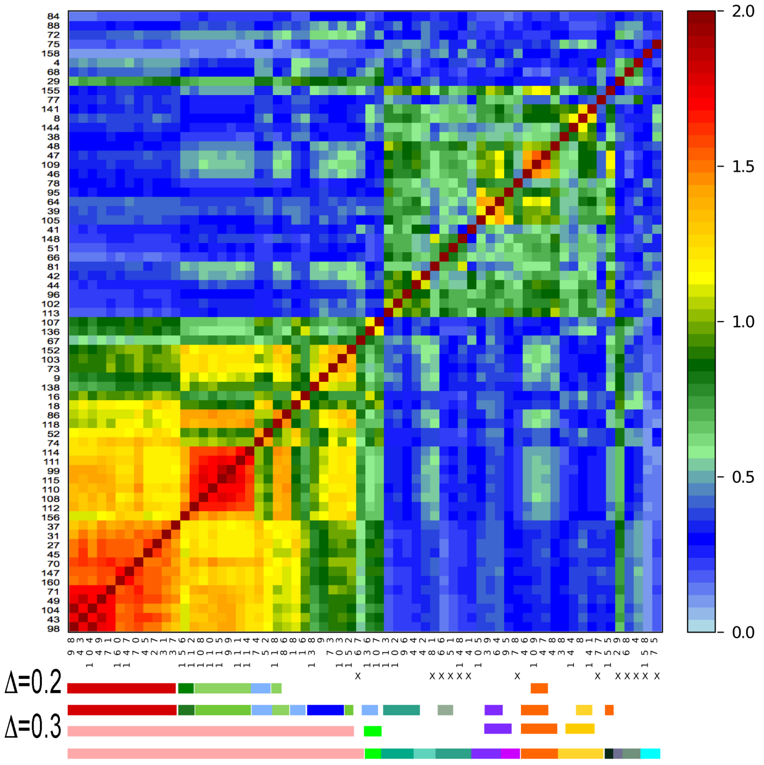

SI Figure 3. Coevolution scores matrix clustered with CLAG without symmetricity condition. Clustering of the MST matrix of coevolution scores for the globin protein family (Baussand and Carbone, *PLoS Computational Biology*, 2009). It is a squared matrix on 67 alignment positions selected by the MST method as susceptible to coevolve. Clustering was realized running CLAG with no symmetricity condition. Slices of the clusterized matrix associated to all key aggregates are obtained with $\Delta = 0.3$ and environmental scores ≥ 0.5. The order of the 67 positions in the y-axis follows the order of the key aggregates positions in the x-axis (from left to right). Positions belonging to key aggregates obtained for $\Delta = 0.2$ (top) and $\Delta = 0.3$ (bottom) with environmental and symmetric scores $= 1$ and < 0.5 respectively are reported at the bottom of the matrix with the help of colored bars. Positions marked by a X are those that do not appear in the clustering of the matrix when the symmetricity condition is used, as illustrated in Figure 6.

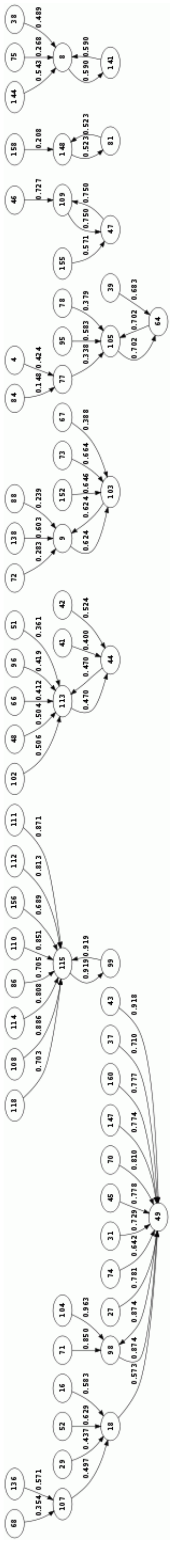

SI Figure 4. SCAP clusters of the globine coevolution matrix.SCAP clusters obtained with $p = 0.13$.

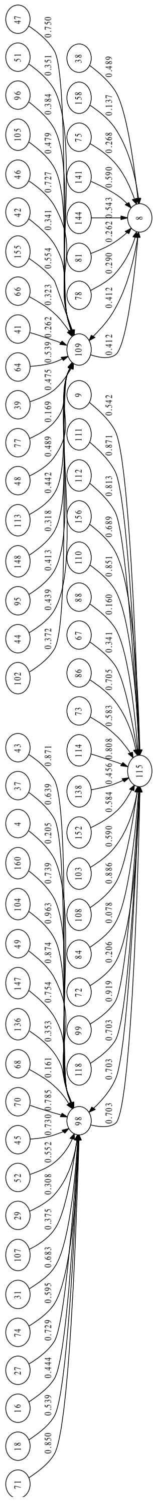

SI Figure 5. SCAP clusters of the globine coevolution matrix.SCAP clusters obtained with $p = 1.0$.

Key aggregates	first		last	
	S_{sym}	S_{env}	S_{sym}	S_{env}
104 147 160 27 31 37 43 45 49 70 71 98	1	1	1	1
108 110 111 112 114 115 156 99	1	1	1	1
109 46 47 48	1	1	0.6	0.94
107 136	0.6	1	0.6	1
141 144 38 8	0.6	1	0.6	0.88
105 39 64 95	0.6	1	0.6	0.67
103 118 138 152 16 18 52 73 74 86 9	0.6	1	0.6	0.52
155	0.6	0.97	0.6	0.97
102 113 42 44 96	0.6	0.97	0.6	0.55

SI Table 6. CLAG globine analysis: rank of key aggregates. Key aggregates are ranked with respect to their scores that represent the strength of the aggregation. Colors correspond to those employed for identifying clusters in Fig. 6. The second and third columns (first) report the scores of the first cluster entering the key aggregate and the last two columns (last) report the scores of the last cluster completing the key aggregate.

6. Synthetic datasets analysis

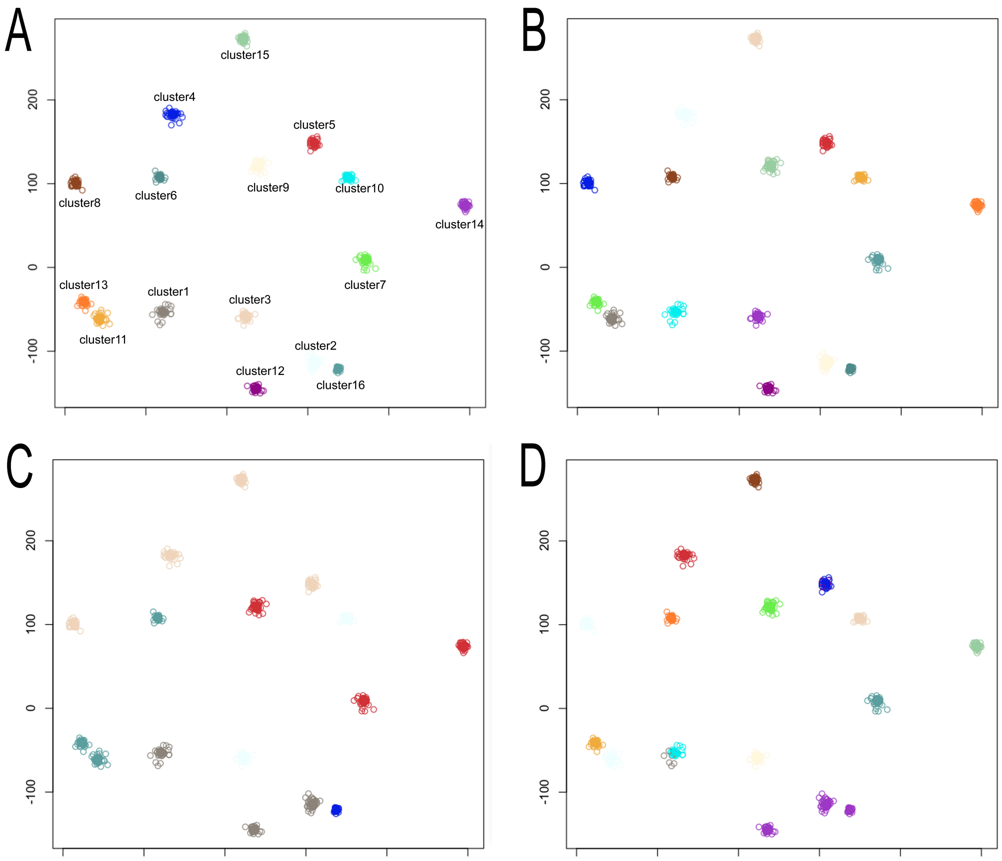

SI Figure 6. Clustering of the synthetic dataset Dim32. A: the 32-dimensional dataset contains 1024 points and 16 clusters generated with a gaussian distribution (http://cs.joensuu.fi/sipu/datasets/). CLAG perfectly distinguished the 16 clusters and it was run with $\Delta = 0.05$ and scores > 0.5. Besides CLAG, different clustering algorithms have been run on this synthetic dataset: c-means (B), MCLUST (C), and k-means (D). k-means and c-means were run with 16 clusters, and MCLUST with "ellipsoidal, unconstrained with 6 components" as best model. For k-means, cluster 1 and cluster 5 are split in several k-means clusters. c-means recognizes the 16 clusters correctly. Figures ABCD are realized by plotting the first two columns of the matrix describing the dataset.

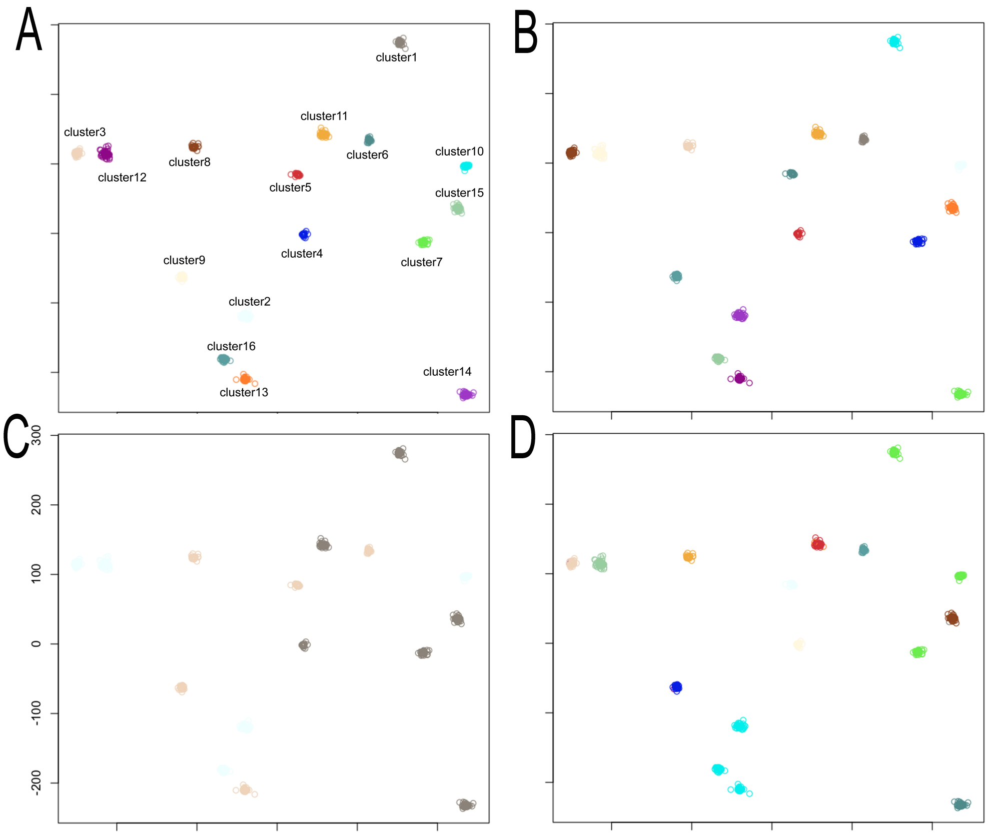

SI Figure 7. Clustering of the synthetic dataset Dim64. A: the 64-dimensional dataset contains 1024 points and 16 clusters generated with a gaussian distribution (http://cs.joensuu.fi/sipu/datasets/). CLAG perfectly distinguished the 16 clusters and it was run with $\Delta = 0.05$ and scores > 0.5. Besides CLAG, different clustering algorithms have been run on this synthetic dataset: c-means (B), MCLUST (C), and k-means (D). k-means and c-means were run with 16 clusters, and MCLUST with "ellipsoidal, unconstrained with 3 components" as best model. For k-means, clusters 3, 9, 11, 15 are split in several k-means clusters. c-means recognizes the 16 clusters correctly. Figures ABCD are realized by plotting the first two columns of the matrix describing the dataset.

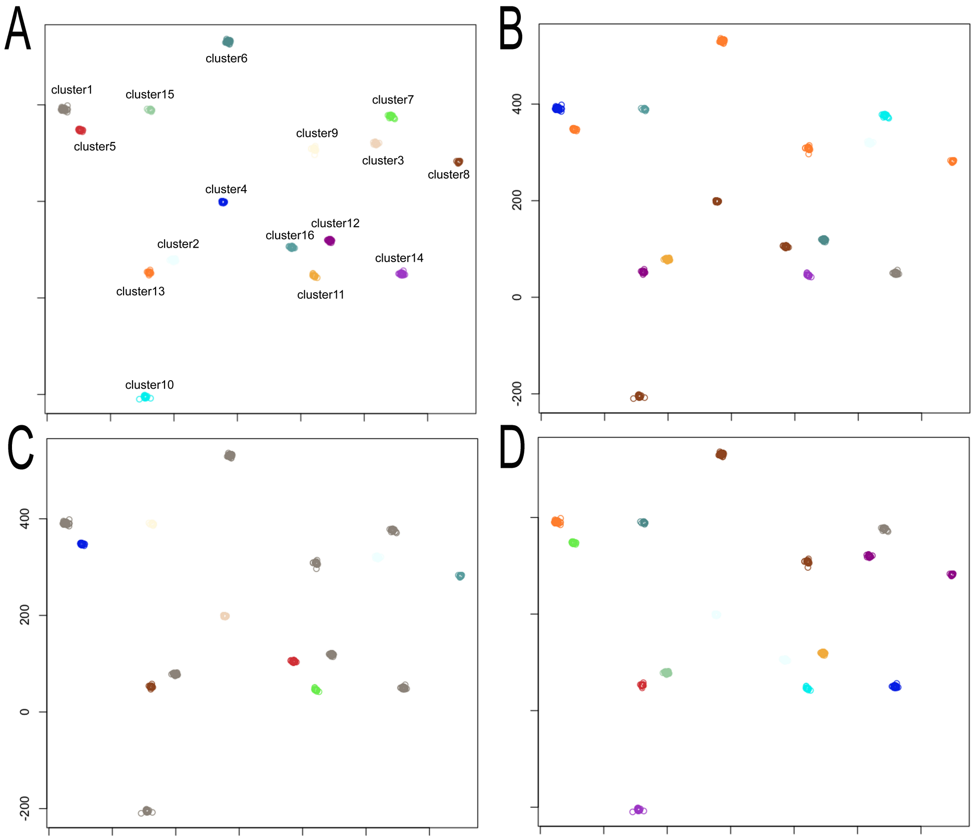

SI Figure 8. Clustering of the synthetic dataset Dim128. A: the 128-dimensional dataset contains 1024 points and 16 clusters generated with a gaussian distribution (http://cs.joensuu.fi/sipu/datasets/). CLAG perfectly distinguished the 16 clusters and it was run with $\Delta = 0.05$ and scores > 0.5. Besides CLAG, different clustering algorithms have been run on this synthetic dataset: c-means (B), MCLUST (C), and k-means (D). k-means and c-means were run with 16 clusters, and MCLUST with "ellipsoidal, equal variance with 9 components" as best model. For k-means, clusters 1, 13 are split in several k-means clusters. c-means clusters the ensemble in only 11 clusters. Figures ABCD are realized by plotting the first two columns of the matrix describing the dataset.

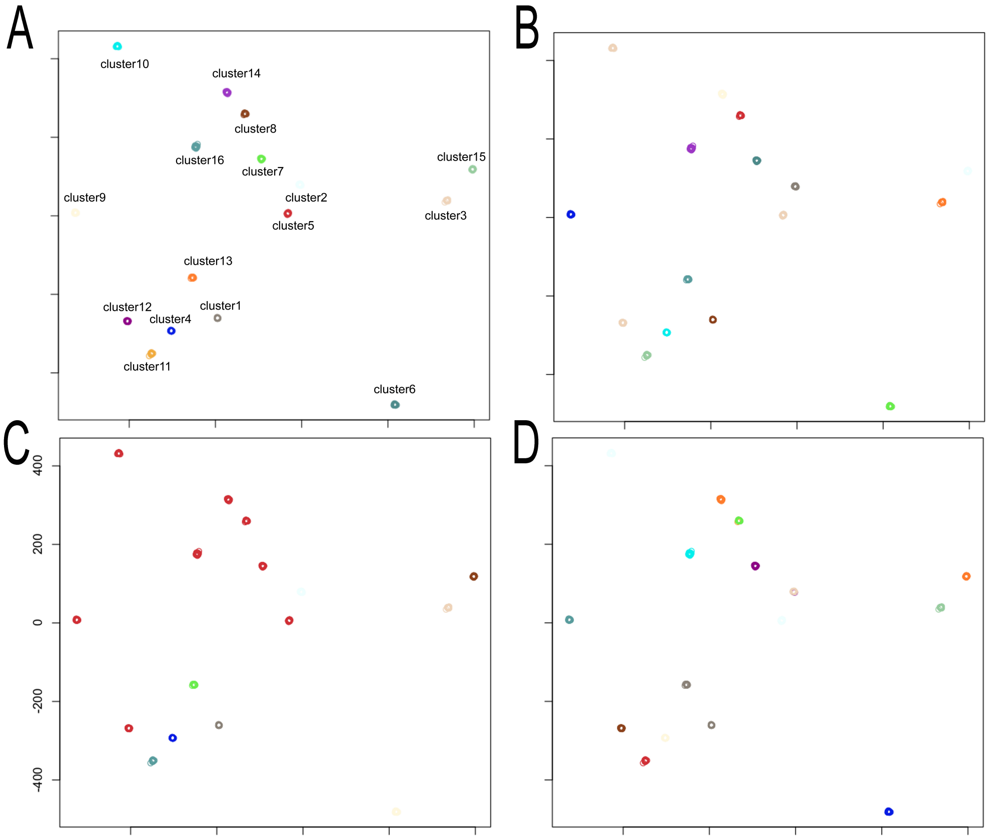

SI Figure 9. Clustering of the synthetic dataset Dim256. A: the 256-dimensional dataset contains 1024 points and 16 clusters generated with a gaussian distribution (http://cs.joensuu.fi/sipu/datasets/). CLAG perfectly distinguished the 16 clusters and it was run with $\Delta = 0.05$ and scores > 0.5. Besides CLAG, different clustering algorithms have been run on this synthetic dataset: c-means (B), MCLUST (C), and k-means (D). k-means and c-means were run with 16 clusters, and MCLUST with “ellipsoidal, equal variance with 9 components” as best model. For k-means, clusters 9, 2, 8 are split in several k-means clusters. c-means clusters the ensemble in only 14 clusters. Figures ABCD are realized by plotting the first two columns of the matrix describing the dataset.

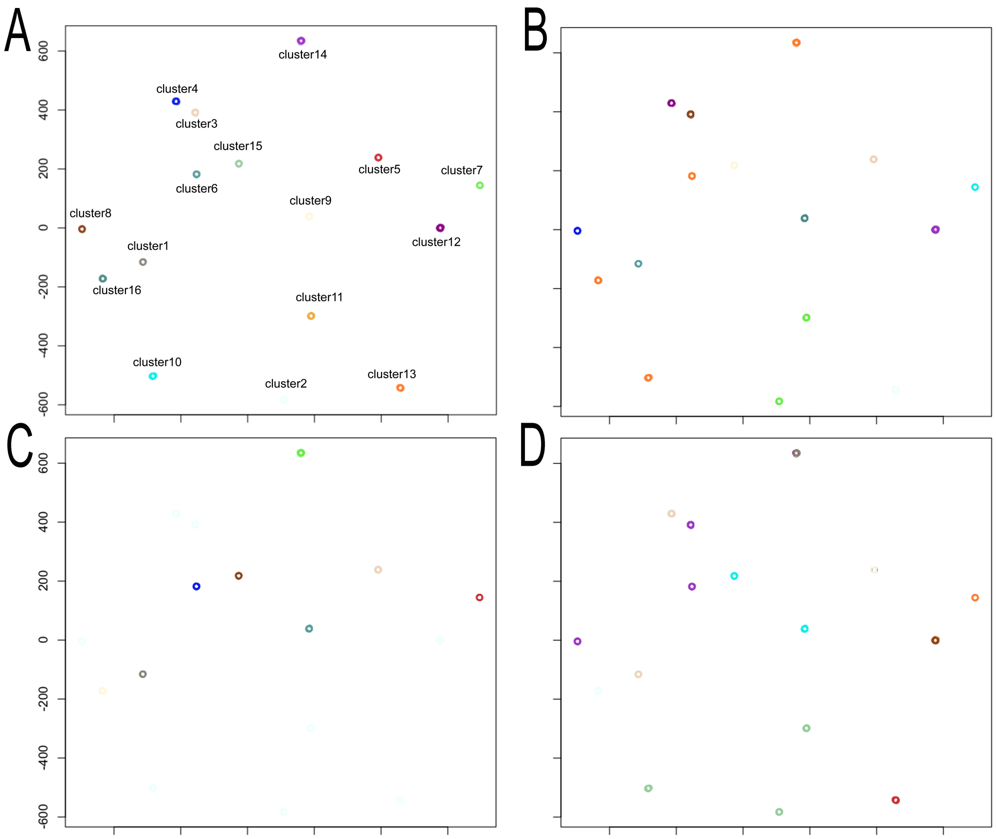

SI Figure 10. Clustering of the synthetic dataset Dim512. A: the 512-dimensional dataset contains 1024 points and 16 clusters generated with a gaussian distribution (http://cs.joensuu.fi/sipu/datasets/). CLAG perfectly distinguished the 16 clusters and it was run with $\Delta = 0.05$ and scores > 0.5. Besides CLAG, different clustering algorithms have been run on this synthetic dataset: c-means (B), MCLUST (C), and k-means (D). k-means and c-means were run with 16 clusters, and MCLUST with "ellipsoidal, equal variance with 9 components" as best model. For k-means, clusters 5, 12, 13, 14 are split in several k-means clusters. c-means clusters the ensemble in only 12 clusters. Figures ABCD are realized by plotting the first two columns of the matrix describing the dataset.

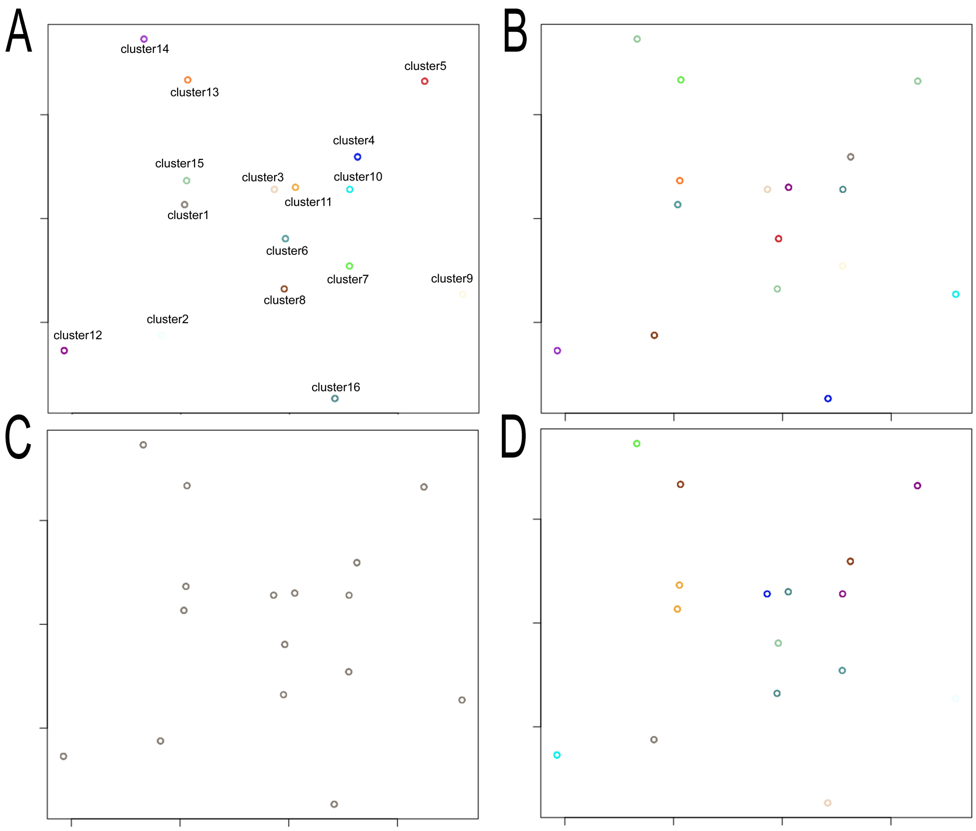

SI Figure 11. Clustering of the synthetic dataset Dim1024. A: the 1024-dimensional dataset contains 1024 points and 16 clusters generated with a gaussian distribution (http://cs.joensuu.fi/sipu/datasets/). CLAG perfectly distinguished the 16 clusters and it was run with $\Delta = 0.05$ and scores ≥ 0.5. Besides CLAG, different clustering algorithms have been run on this synthetic dataset: c-means (B), MCLUST (C), and k-means (D). k-means and c-means were run with 16 clusters, and MCLUST with "ellipsoidal multivariate normal with 1 component" as best model. For k-means, clusters 3, 6, 7 are split in several k-means clusters. c-means clusters the ensemble in only 14 clusters. Figures ABCD are realized by plotting the first two columns of the matrix describing the dataset.

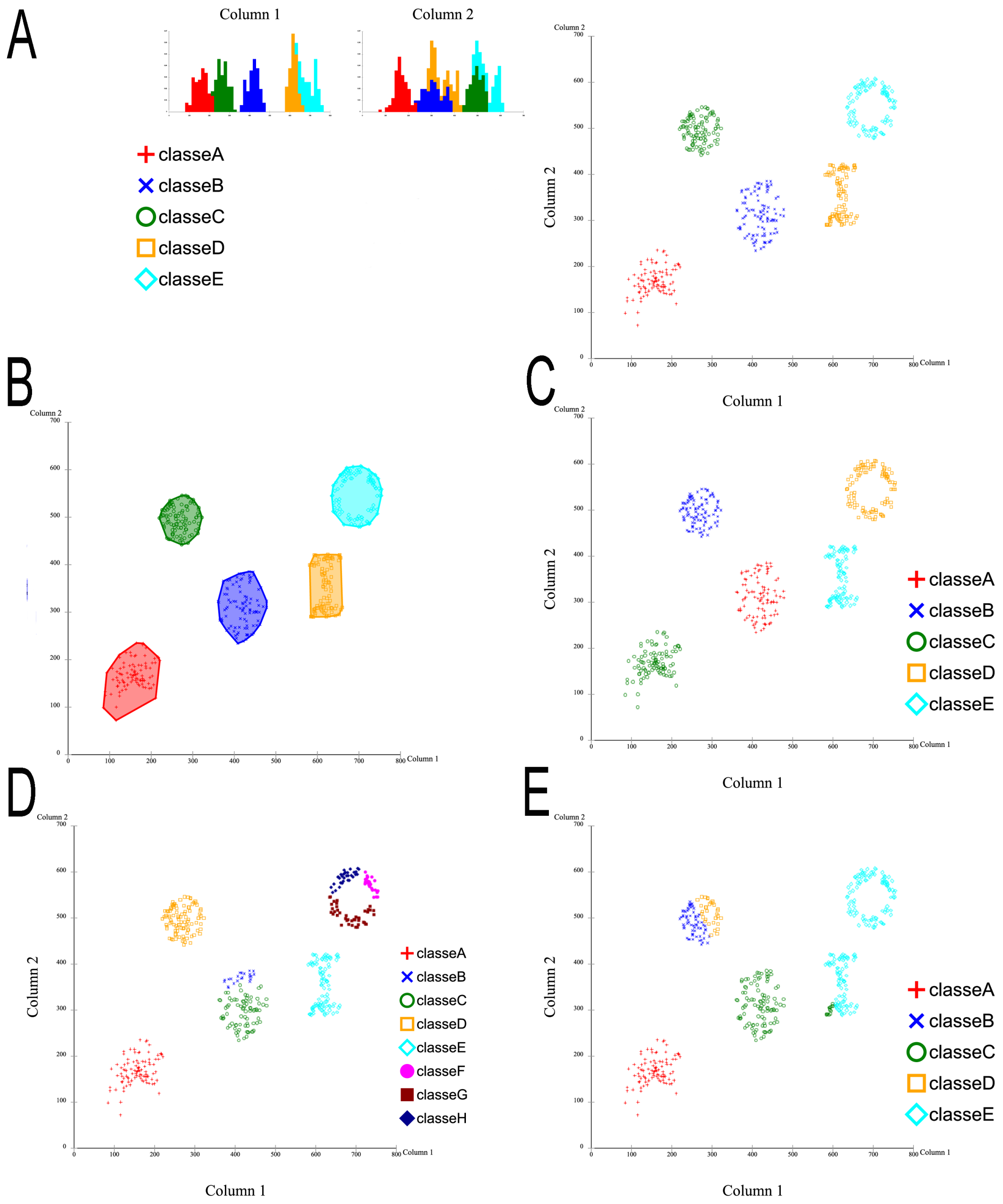

SI Figure 12. Clustering of the synthetic dataset G4.A: the 2D dataset contains 500 points and 5 clusters generated with difficulty level=1 and density level= 3, by using the software DataGenerator.jnlp, downloadable at `http://webdocs.cs.ualberta.ca/ yaling/Cluster/Php/index.php`. Different clustering algorithms have been run on this synthetic dataset: CLAG (B), k-means (E), c-means (C) and MCLUST (D). CLAG was run with $\Delta = 0.05$ and scores $= 1$, k-means and c-means with 5 clusters, and MCLUST with "ellipsoidal, unconstrained" as best model and with 8 components.

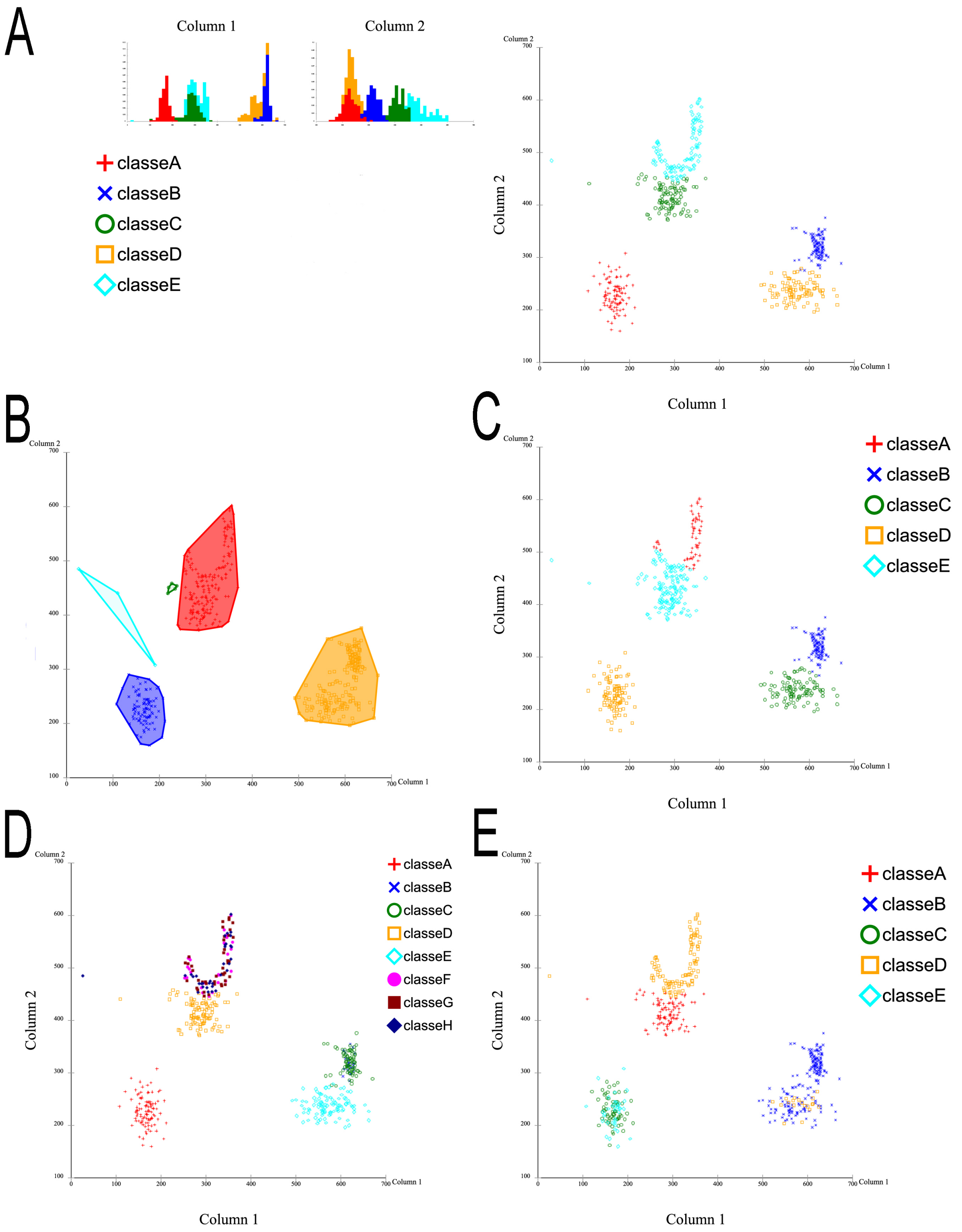

SI Figure 13. Clustering of the synthetic dataset G5.A: the 2D dataset contains 500 points and 5 clusters generated with difficulty level=2 and density level= 3, by using the software DataGenerator.jnlp, downloadable at `http://webdocs.cs.ualberta.ca/ yaling/Cluster/Php/index.php`. Different clustering algorithms have been run on this synthetic dataset: CLAG (B), k-means (E), c-means (C) and MCLUST (D). CLAG was run with $\Delta = 0.05$ and scores $= 1$, k-means and c-means with 5 clusters, and MCLUST with "diagonal, varying volume and shape" as best model and with 8 components.

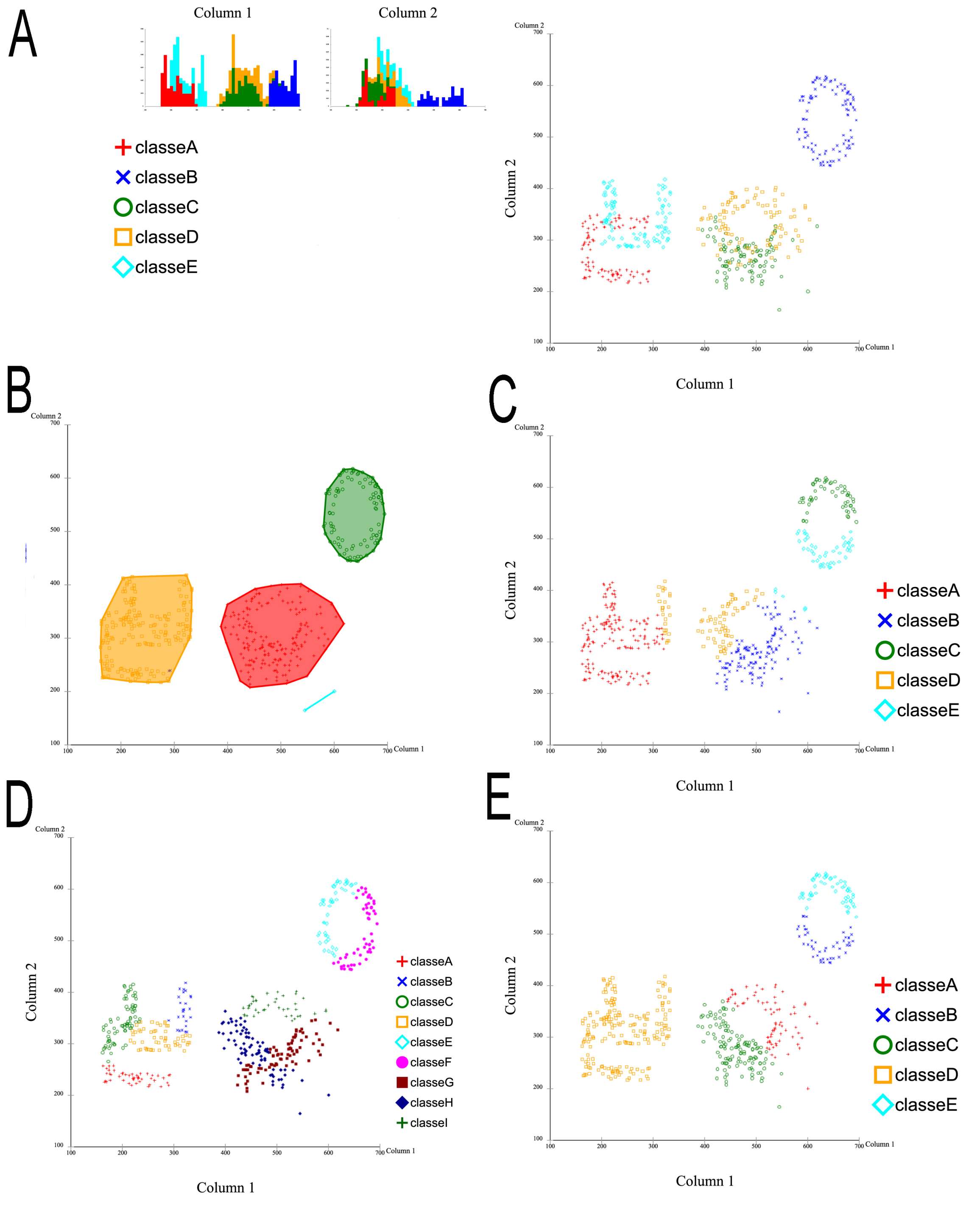

SI Figure 14. Clustering of the synthetic dataset G6.A: the 2D dataset contains 500 points and 5 clusters generated with difficulty level=2 and density level= 3, by using the software DataGenerator.jnlp, downloadable at `http://webdocs.cs.ualberta.ca/ yaling/Cluster/Php/index.php`. Different clustering algorithms have been run on this synthetic dataset: CLAG (B), k-means (E), c-means (C) and MCLUST (D). CLAG was run with $\Delta = 0.05$ and scores $= 1$, k-means and c-means with 5 clusters, and MCLUST with "ellipsoidal, equal volume and shape" as best model and with 9 components.

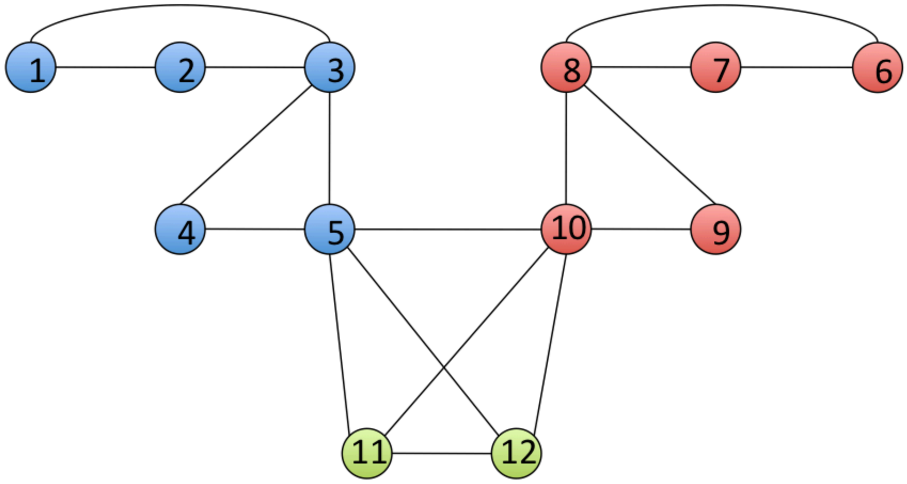

SI Figure 15. Aggregation graph of the toy example. The aggregation graph constructed with CLAG aggregation step applied to the affine clusters described in the toy example. The blue aggregate is constructed first (from clusters C_1 and C_2), the red one is constructed next (from clusters C_3 and C_4), and finally the two aggregates (blue and red) are put together with cluster C_5 that generates a third aggregate (colored green), linking both the blue and the red aggregates. Notice that each original cluster C_i is associated to a clique.
